# Magma as a carrier of non-stoichiometric oxygen linking Earth’s interior and atmosphere

**DOI:** 10.1093/nsr/nwag539

**Published:** 2026-08-25

**Authors:** Yongjiang Xu, Haiyang Niu, Wim van Westrenen, Ruiheng Liu, Peiyan Wu, Ho-Kwang Mao, Yanhao Lin

**Affiliations:** Center for High Pressure Science and Technology Advanced Research, Beijing 100193, China; Lin Earth and Planetary Laboratory, Center for High Pressure Science and Technology Advanced Research, Beijing 100193, China; State Key Laboratory of Solidification Processing, International Centre for Materials Discovery, School of Materials Science and Engineering, Northwestern Polytechnical University, Xi’an 710072, China; Lin Earth and Planetary Laboratory, Center for High Pressure Science and Technology Advanced Research, Beijing 100193, China; Department of Earth Sciences, Faculty of Science, Vrije Universiteit Amsterdam, Amsterdam 1081 HV, The Netherlands; State Key Laboratory of Solidification Processing, International Centre for Materials Discovery, School of Materials Science and Engineering, Northwestern Polytechnical University, Xi’an 710072, China; Center for High Pressure Science and Technology Advanced Research, Beijing 100193, China; Lin Earth and Planetary Laboratory, Center for High Pressure Science and Technology Advanced Research, Beijing 100193, China; Center for High Pressure Science and Technology Advanced Research, Beijing 100193, China; Shanghai Advanced Research in Physical Sciences, Shanghai 201203, China; Center for High Pressure Science and Technology Advanced Research, Beijing 100193, China; Lin Earth and Planetary Laboratory, Center for High Pressure Science and Technology Advanced Research, Beijing 100193, China

**Keywords:** excess oxygen, high-pressure experiments, oxygen-rich deep magmas, oxygen cycle, atmospheric oxygenation

## Abstract

Magma generation and transport govern planetary evolution. Although oxygen is the most abundant anion in Earth’s silicate interior, its role in magma is conventionally ignored, with magma oxygen contents assumed to be simply fixed by cation stoichiometry. Here we show, using high-pressure and high-temperature experiments supported by molecular dynamics simulations, that silicate magma can incorporate substantially more oxygen than permitted by traditional valence-state constraints. Excess oxygen acts as a previously unrecognized volatile component, increasing in concentration from near zero at 1 atm to > ∼5 wt.% above 5 GPa, more than five times the maximum oxygen increase attainable in Earth’s solid mantle through iron redox changes. As oxygen-rich magmas ascend and decompress, their capacity to retain excess oxygen decreases, driving the release of O_2_. This behaviour provides a direct mechanism with the potential to link mantle melting to oxidation of Earth’s shallow interior and atmosphere, and identifies magma as a dynamic reservoir in planetary oxygen cycles.

## Teaser

Magma can store more oxygen than previously thought and release it as it rises, providing a new link between Earth’s deep interior and the oxidation of its surface and atmosphere.

## INTRODUCTION

Oxygen dominates the silicate Earth, comprising more than half of its atoms and most of the volume of rock-forming minerals [1]. In addition to its important role in the atmosphere and in the habitability of Earth’s surface, its interior distribution is thought to be a key regulator of planetary differentiation and mantle dynamics through its effect on interior redox state. Variations in the redox state of the mantle and crust are traditionally interpreted through changes in the valence of multivalent cations such as iron [2,3] or vanadium [4], implicitly assuming that oxygen concentrations in minerals and melts are fixed by stoichiometric constraints. Whether silicate melts themselves can store oxygen as an independent volatile component, however, remains largely unexplored. This question is fundamental because melts are the principal agents of chemical transport between Earth’s deep interior and its surface.

Geological records indicate that atmospheric oxygen levels rose episodically during Earth history, most prominently during the Great Oxygenation Events [5,6]. These increases broadly coincide with episodes of extensive mantle melting and the emplacement of large igneous provinces (LIPs) [7,8], suggesting a possible link between deep magmatism and surface oxidation. However, the physical basis for such a connection is unclear [9,10]. Existing explanations invoke indirect processes such as recycling of surface-derived volatiles, pressure-release melting, mantle temperature variations and long-term redox evolution of mantle reservoirs [11–15]. Whether mantle melting itself can directly generate and transfer oxidizing power to Earth’s surface therefore remains unresolved.

Addressing this question requires understanding of how oxygen is stored and transported during melting. Oxygen is the dominant anion in silicate melts, but the traditional assumption that its abundance is fixed by cation valence balance, suggests that oxygen has no independent role as a volatile or mobile component. If this traditional assumption fails at high pressure, melts could act as transient reservoirs of oxidizing power within the mantle.

Here, we show, using high-temperature–pressure experiments with direct oxygen abundance measurements supported by molecular dynamics simulations, that silicate melts can incorporate substantial excess oxygen as a previously unrecognized volatile component beyond traditional stoichiometric limits. We conducted high-temperature–pressure experiments at 1 atm to 7 GPa and 1200 ± 5 to 1800 ± 5°C on a mid-ocean ridge basalt (MORB) composition [16], using large-volume high-pressure apparatus (piston cylinder and cubic press, jointly referred to as LVP) and a high-temperature furnace at HPSTAR Beijing, with PtO_2_ as the oxygen source. The excess oxygen (EO; given in wt.%) is the percentage of oxygen in samples after assigning stoichiometric proportions of oxygen to the measured concentrations of all elements with positive valence states and subtracting the sum of these proportions from the directly measured O abundances. To acquire accurate abundance measurements of all elements, including hydrogen and carbon, both a standard electron probe micro-analyser and secondary ion mass spectrometry (SIMS) were used. To corroborate our experimental observations and probe the atomic-scale mechanism(s) of EO incorporation in silicate melts, we also performed molecular dynamics (MD) simulations of the Mg–Si–O system using deep neural network (DNN) interatomic potentials with *ab initio* accuracy. Full details of starting material synthesis, experimental setups and conditions, properties of run products, calibration of oxygen concentration measurements and the MD simulations are provided in the Materials and methods and Supplementary data sections.

## RESULTS AND DISCUSSION

**Figure 1.**
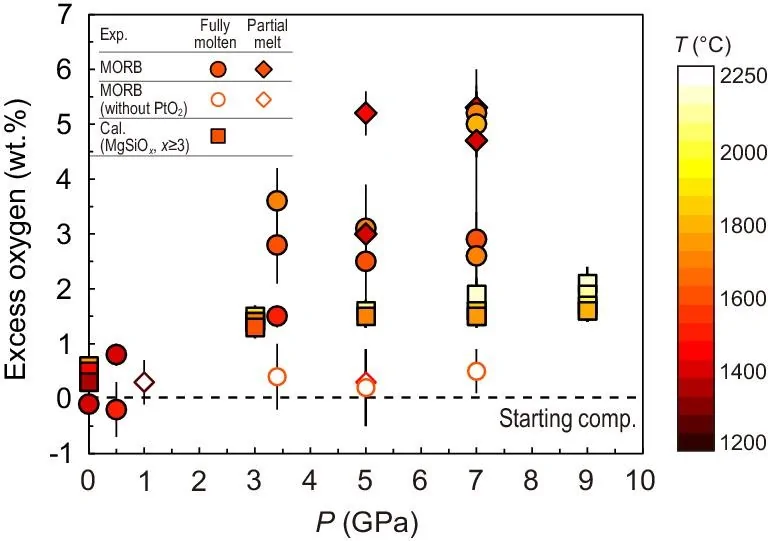
EO in silicate glass as a function of pressure. Circles and diamonds represent results of experiments with the MORB starting glass; squares represent results of molecular dynamics calculations in the MgSiO_x_-O_2_ (*x* ≥ 3) system. Filled and open circles and diamonds represent fully and partially molten materials from experiments, respectively. The EO of both experimental MORB and simulated MgSiO_3_ starting compositions is indicated by a dotted line. The symbols are colored for temperature according to the scale bar. Data are provided in Tables 1 and 2.

Calculated EO values are summarized in Table 1 and shown as a function of pressure in Fig. 1. For these EO calculations, we conservatively assume that all iron in the glasses has a valence of +3. EO values in the large-volume high-pressure (LVP) experiments yielding fully molten MORB (filled circles in Fig. 1) systematically increase with pressure from near zero (−0.1 ± 0.2 wt.%) at 1 atm to 5.2 ± 0.2 wt.% at 7 GPa. LVP experiments that were run at identical high-pressure, high-temperature conditions but without an additional oxygen source yield substantially lower EO values between 0.2 ± 0.7 and 0.5 ± 0.4 wt.%, further confirming that magma at high pressure can store EO. EO values of glass in partially molten MORB experiments are generally higher than EO values of glass in fully molten MORB. This suggests that none of the LVP experiments reached EO saturation under these conditions, that maximum EO values could be even higher than those reported in Fig. 1 and/or that temperature may also affect EO. EO values do not show any correlation with the measured glass concentrations of CO_2_, H_2_O (Fig. S1) or major elements (Fig. S2). It is worth noting that the EO concentrations reported here represent the minimum oxygen storage capacity preserved in quenched glasses. Some EO may have degassed during the temperature quenching process. Quantifying the kinetics and magnitude of EO loss during quenching will require future *in situ* investigations. Overall, Fig. 1 shows that the standard practice in geoscience of deriving melt O contents indirectly from measurements of cation abundances can be highly inaccurate, underestimating true melt O abundance by up to >5 wt.% under oxidizing condition.

**Table 1.**
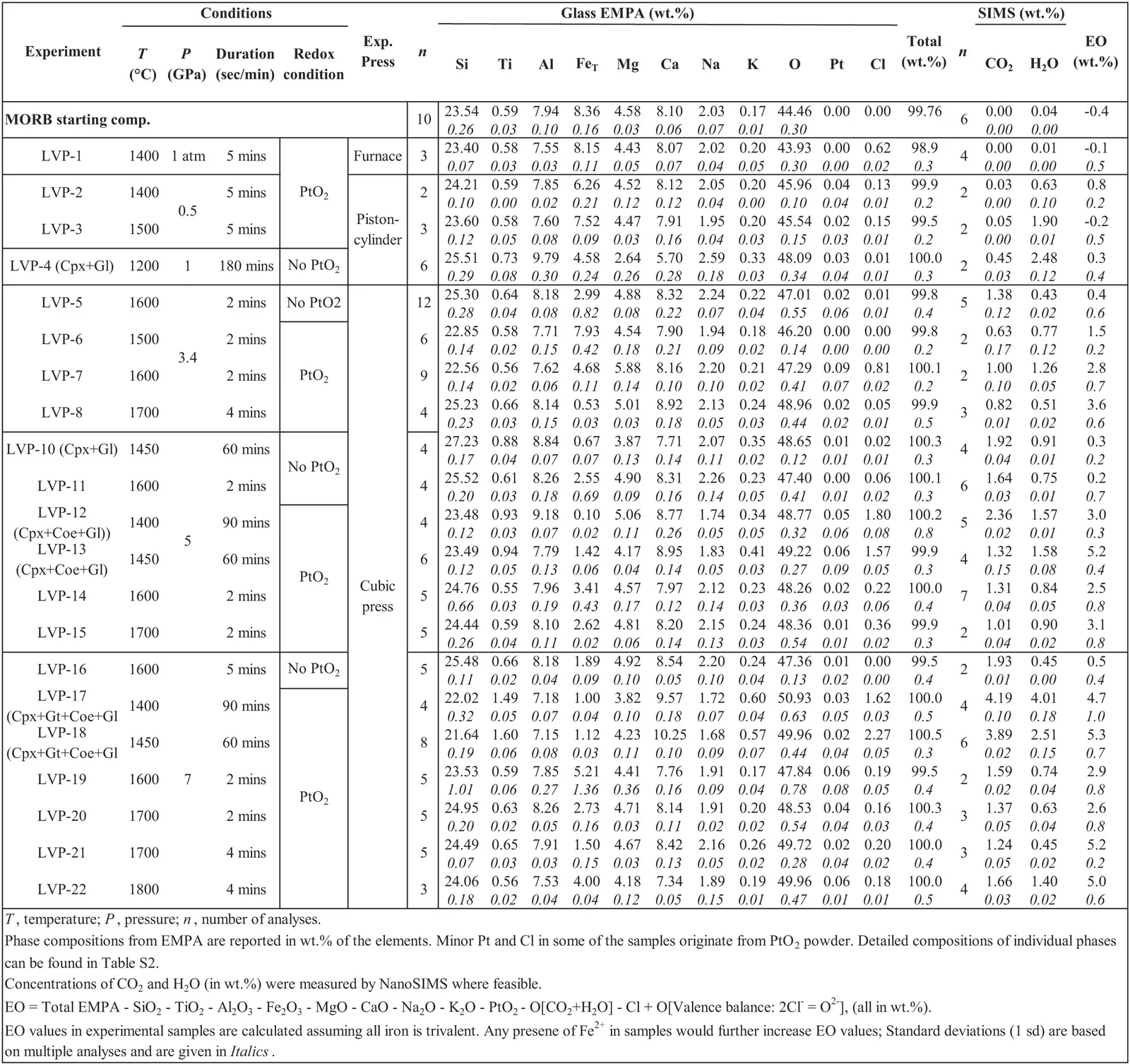
Summary of experimental results.

**Figure 2.**
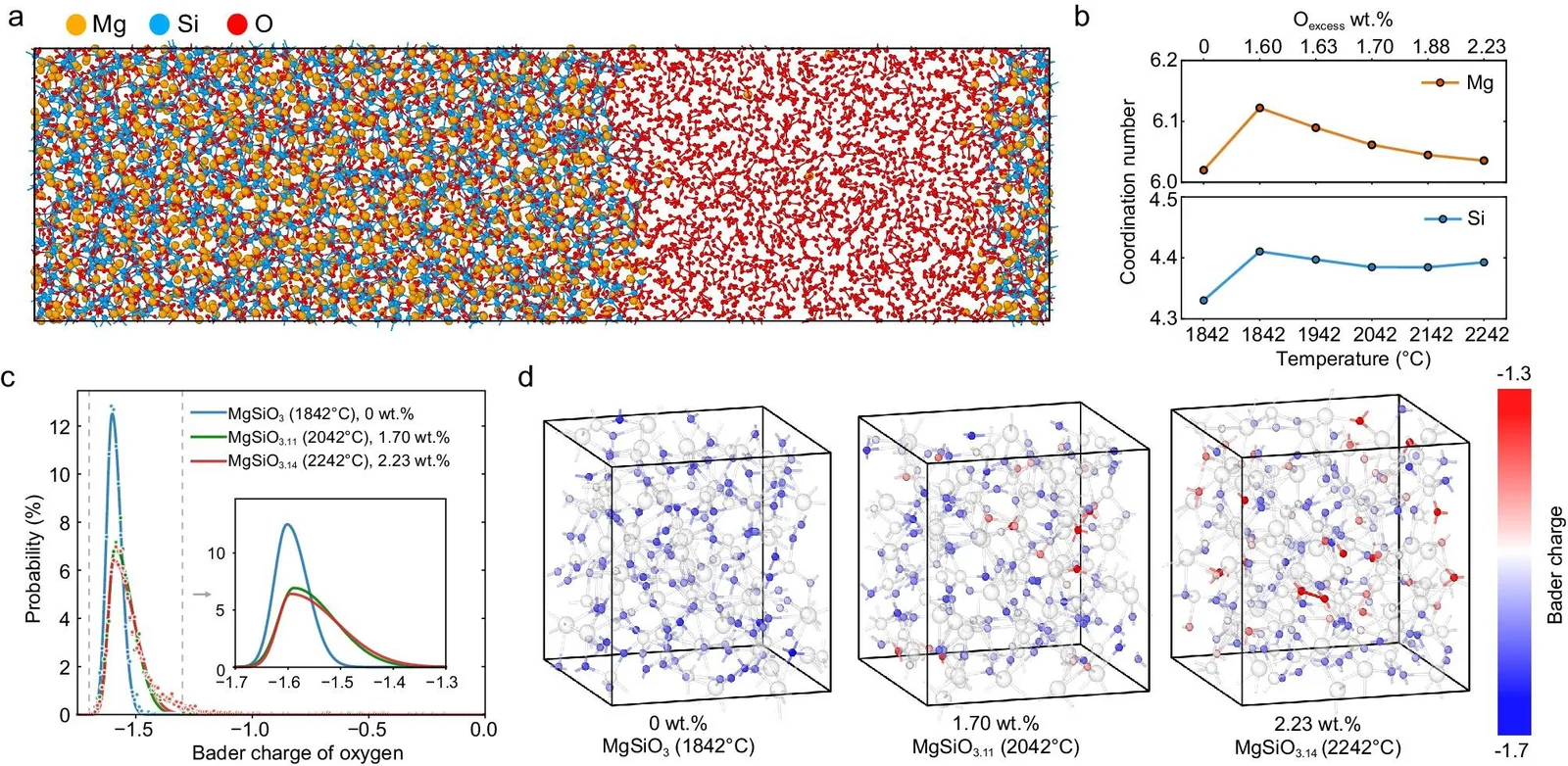
Structure and electronic signatures of oxygen-excess MgSiO_x_ (*x* > 3) melts. (a) Representative coexistence configuration of liquid MgSiO*_x_* and oxygen at 9 GPa and 1842°C, showing continuous oxygen diffusion into the silicate melt until the thermodynamic equilibrium state is reached. Mg, Si and O atoms are identified in the figure legend. (b) Average coordination numbers of Mg and Si atoms as a function of temperature and excess oxygen content. At 1842°C, the coordination numbers of Mg and Si increase from 6.02 and 4.33, respectively, in stoichiometric MgSiO_3_ to 6.12 and 4.41, respectively, in oxygen-excess MgSiO_x_. With increasing temperature, the melt becomes more oxygen-excess, accompanied by decreasing Mg coordination and increasing Si coordination, consistent with preferential bonding of excess oxygen to Si. The cut-off radii are determined by the first minima of the radial distribution functions (RDFs): 2.865 Å for Mg and 2.325 Å for Si. (c) Bader charge distributions of oxygen under varying oxygen contents, showing progressive broadening toward higher charge states as the melt becomes more oxygen-excess. (d) Oxygen charge distributions across three compositions, illustrating a growing population of oxygen atoms with charges > −1.5 as the melt becomes more oxygen-excess. Mg and Si atoms are rendered transparently for clarity.

To further understand these experimental results and provide an atomistic view, we modelled oxygen-rich MgSiO*_x_* liquids (*x* ≥ 3) as a proxy for basaltic compositions at 1 atm to 9 GPa and 1352–2242°C using MD simulations performed with LAMMPS [17]. These simulations were driven by a DNN potential, developed iteratively from previous work [18,19], with *ab initio* accuracy. Two-phase coexistence simulations between molten MgSiO_3_ and pure O_2_ show continuous diffusion of oxygen from the O_2_ reservoir into liquid MgSiO_3_ until chemical equilibrium is reached (Figs 2a and S3), demonstrating that liquid oxygen-excess MgSiO_x_ compositions are thermodynamically favored at high pressure. The equilibrium EO content was obtained by averaging over the final 2 ns of the trajectories for each *P*–*T* condition. The simulated EO content increases from 0.33 ± 0.17 wt.% at 1 atm to 2.12 ± 0.26 wt.% at 9 GPa (Table 2), providing atomistic support for both the EO values measured in our high-pressure–temperature experiments and the increase in EO with increasing pressure (Fig. 1).

**Table 2.**
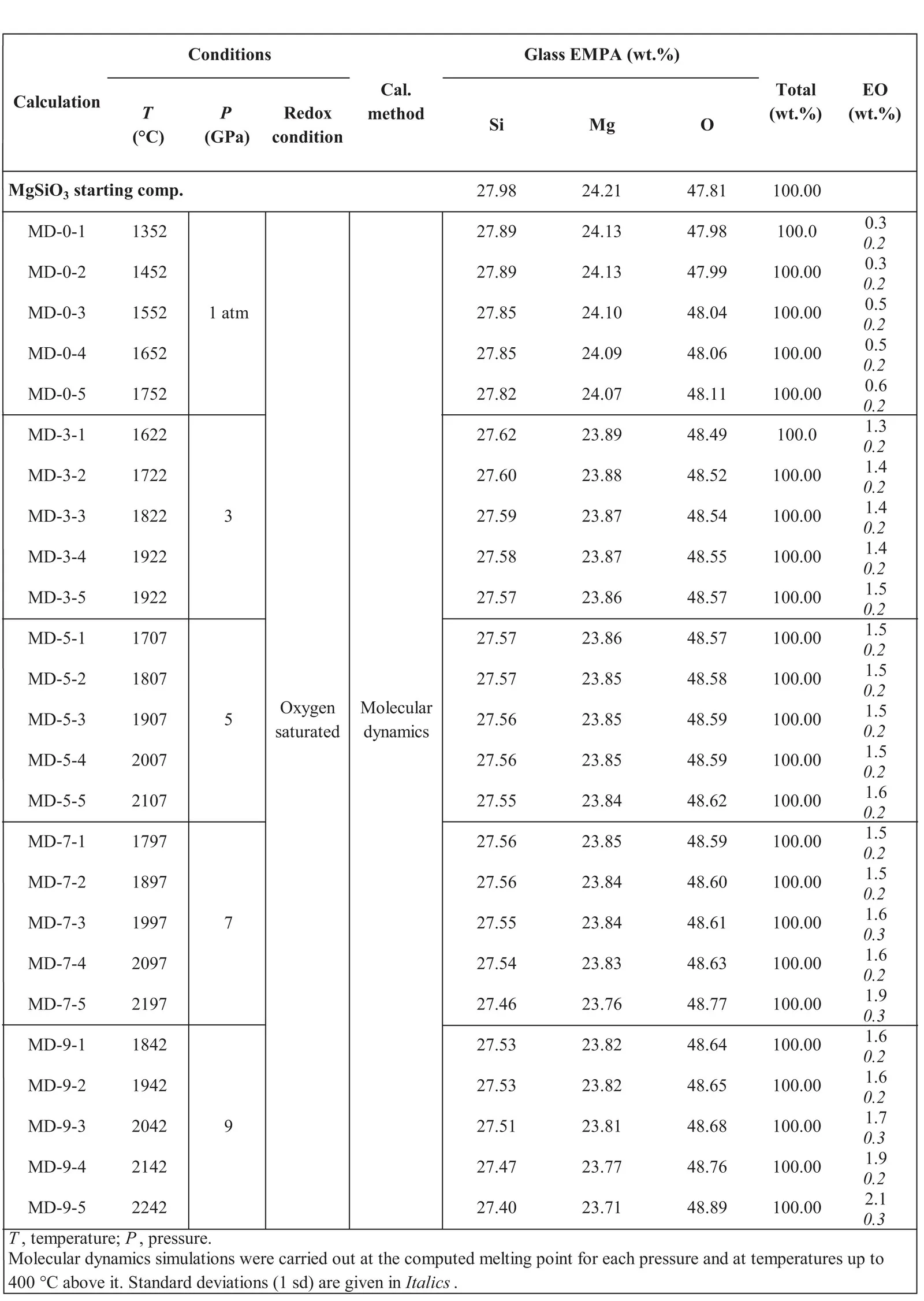
Summary of simulated results.

To evaluate how EO modifies melt structure and bonding, we analysed the coordination and electronic environments in MgSiO_x_ liquids at 9 GPa and 1842°C and compared them with stoichiometric MgSiO_3_ at the same conditions (Fig. 2a-d). In oxygen-excess MgSiO*_x_* (*x* > 3), an increase in oxygen content is accompanied by a larger relative increase in Mg coordination and a slighter relative increase in Si coordination (Fig. 2b). As the temperature rises, the Mg coordination number decreases, while the Si coordination number shows a continued upward trend. This indicates that EO preferentially bonds to Si as temperature increases. Bader charge analyses further reveal that oxygen atoms shift towards less negative valences (> −1.5*e*) and exhibit more broadly distributed charge states as the melt becomes more oxygen-rich, whereas Mg retain nearly constant charge states and Si becomes slightly less charged (Fig. 2c and d, and Fig. S4). This observation is consistent with the coordination trends of the constituent elements and provides insight into how EO can be accommodated in MgSiO*_x_* beyond the conventional picture of isolated oxide O^2−^ anions. At elevated temperatures, a small fraction of O atoms, 1.1% at 9 GPa and 2242°C, exhibits short O–O distances and effective charges on the O atoms that are less negative than −1*e*, indicating the formation of peroxide-like ${\mathrm{O}}_2^{2 - }$ species under oxygen-rich conditions. Although such peroxide-like states may appear counter-intuitive in Mg silicates, similar O–O bonded peroxide species have previously been reported in thermodynamically stable aluminum oxides and Mg silicates under high-pressure conditions [20,21].

Raman spectra of quenched silicate glass from LVP samples do not show O_2_ peaks (Fig. S5), suggesting that the EO is incorporated structurally into the silicate glass, rather than in the form of O_2_ bubbles. This further confirms that the quenched melts remained below oxygen saturation. The MORB glass Raman spectra in Fig. S5 show distinct bands at ∼400–650 and ∼800–1200 cm^−1^ from the modes of M (Si, Mg, Ca, Al, Fe)–O bonding in glass [22,23]. The changes in Raman band frequencies and intensities could be attributed to pressure-induced structural distortion [24]. The Raman peak at ∼1100 cm^−1^ is attributed to dissolved carbonate (CO_3_)^2−^ in the silicate glass [25], rather than to EO. No additional peaks were identified in any of the samples containing excess O molecules, suggesting that the Raman shift associated with cation−EO bonding overlaps with the modes of M (Si, Mg, Ca, Al, Fe)–O bonding.

The increase in EO with increasing pressure from our experiments (Fig. 1) and thermodynamic calculations (Fig. 2) is reminiscent of increases in the solubility of volatile compounds such as water and CO_2_ in silicate magma with increasing pressure [26,27]. The measured wt.% levels of EO suggest that oxygen should be considered as a separate component in silicate melts and should not be assumed to be present in stoichiometric proportions calculated from cation abundances and cation valence state measurements. High-pressure experiments have previously shown that several peroxide solid phases can be stable at deep Earth mantle conditions, including MgO_2_ [28], FeO_2_ [29], CaO_3_ [30] and KO_3_ [31]. This suggests that at these conditions, the effective charge of at least part of the oxygen atos, deviated substantially from the formal oxidation state of O^2−^, can be significantly more positive than the canonical value of −2 [32]. Our MD simulations show that this is also the case in silicate melts at much lower pressures than observed in solid phases.

Recent data from mineral physics, seismology and geochemistry have provided abundant evidence that the oxygen distribution in Earth’s interior is heterogeneous, with the mantle locally containing more reduced and more oxidized domains that show vertical, lateral and temporal variations in redox state [29,32–42]. Locally oxidized regions in the mantle that could provide a source for EO in high-pressure magmas can be formed by many different mechanisms, including (i) incorporation of recycled surface-derived oxidized materials [33,34]; (ii) disproportionation of Fe^2+^ to Fe^3+^ and Fe^0^ either at the magma ocean stage of Earth evolution or subsequently in the solid phase, with the removal of iron droplets to the core [32,35]; (iii) the continued presence of residual primitive oxidized mantle material, which has not been homogenized [39] due to the extremely low diffusion rate of oxygen [40]; (iv) decomposition of goethite or reaction of water and iron in the core to form pyrite-structured iron peroxide FeO_2_ and FeO_2_H*_x_* with removal of hydrogen [29,41,42]; (v) the decomposition of CO_2_ [43]; and (vi) formation of superionic oxygen-deficient davemaoite at the early Earth magma ocean solidification, providing extra oxygen and contributing to oxidation of the deep mantle [44].

The addition of PtO_2_ in our experiments imposes highly oxidizing conditions and represents an oxidizing end-member case, rather than representing average terrestrial mantle conditions. Similar to H_2_O- or CO_2_-solubility experiments, the objective of our study is to establish whether silicate melts can incorporate oxygen as an independent chemical component and, if so, through which mechanism(s) and to what extent. The highest EO contents reported here therefore represent the potential capacity of silicate melts under oxygen-rich conditions, rather than typical EO abundances in mantle-derived magmas.

Our results indicate that basaltic melts formed in locally oxidized reservoirs in the mantle of Earth have a capability to incorporate large amounts of EO. We do not suggest that all mantle-derived magmas contain significant EO. Instead, EO-rich magmas are expected where melting occurs within, or where melts interact with, oxygen-rich mantle reservoirs. The discovery of EO in melt also provides a novel mechanism to explain changes in melting behaviour (e.g. changes in solidus and liquidus temperature) of silicate rocks as a function of oxygen fugacity at elevated pressure. EO is expected to behave as an incompatible element during partial melting. Analogous to the effect of other incompatible elements on melting temperatures, the expectation would be that melting at higher oxygen abundances would occur at lower temperatures than melting at low oxygen abundances, and this is exactly what is observed [13,45–47].

**Figure 3.**
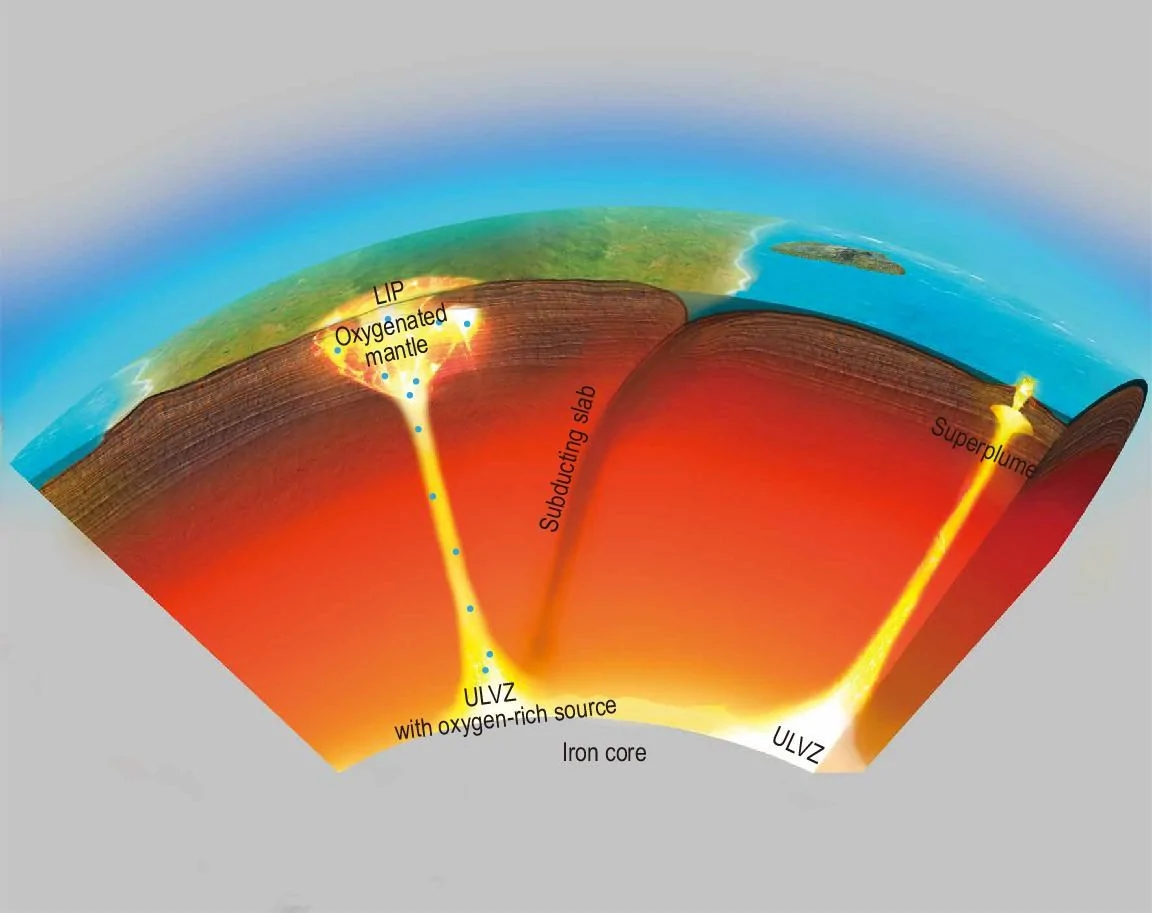
Schematic cross-section of Earth’s mantle illustrating excess oxygen release during ascent large igneous province (LIP) magmas. A deep-seated, oxygen-rich mantle region (left) that may form through several mechanisms discussed in the text [29,32–35,39–44] undergoes enhanced partial melting [13], generating high-volume magmas containing substantial excess oxygen. In contrast, a relatively reduced plume (right) produces much less magma under the same pressure/temperature conditions. As oxygen-rich magmas ascend, forming a LIP at the surface, their capacity to retain excess oxygen decreases to zero, causing O_2_ (represented by dots) to be released from the melt. Such degassing provides a direct mechanism for oxidizing Earth’s upper mantle, crust and atmosphere. This mechanism offers a potential explanation for the temporal coincidence between major atmospheric oxygenation events and episodes of LIP emplacement.

Upon transportation towards the surface, and corresponding decompression, the trends shown in Fig. 1 indicate that at pressures below 10 GPa, oxygen-rich magmas generated from locally oxidized mantle reservoirs will gradually release EO as the EO capacity decreases. This provides a potential mechanism to oxidize the shallow mantle and crust (Fig. 3) and could contribute to explanations for the observed temporal overlap between the formation of some of the largest LIPs and Great Oxidation Events [6]. For example, at least 7.2 Mkm^3^ (million km^3^) LIP magma is estimated to have formed in the interval ∼2.5–2.0 Ga based on geological data [7,8], including ∼41 recognized LIPs, average extrusive area of ∼0.18 Mkm^2^, assumed average thickness of 1 km and an estimated extrusive/intrusive volume ratio of 5%. Considering a basalt density of ∼ 3010 kg/m^3^ as an average density of LIPs [48], ∼0.27 wt.% of EO originating from LIPs formed at ∼2.5–2.0 Ga would need to be released to produce all of the oxygen present in our current atmosphere. This calculation is intended only as a first-order scaling exercise, not a quantitative model for the origin of atmospheric oxygen. Rather, it illustrates that the enormous volumes of LIP magmas could generate a geochemically significant oxidizing flux even if only trace amounts of EO were released.

Although solid-state mantle convection remains the dominant mechanism for mass transport within Earth’s interior, magmatism provides the principal pathway for transporting incompatible components and volatiles from the mantle to the surface. Our results redefine the role of magma in planetary evolution by showing that silicate melts can store and transport substantial oxidizing power independently of conventional redox constraints. Pressure-stabilized EO provides a direct pathway linking mantle melting to atmospheric oxidation, suggesting that episodes of large-scale magmatism may have actively regulated the redox state of Earth’s surface environment. Further, the capacity of deep melts to transiently sequester and release oxygen offers a new perspective on magma ocean evolution, mantle differentiation and volatile cycling on terrestrial planets. Recognizing magma as an engine of planetary oxidation may establish a framework for understanding how interior dynamics help shape atmospheric composition, surface conditions and long-term planetary habitability.

## MATERIALS AND METHODS

A brief description of the experimental and computational methods is provided below; full details are given in the Supplementary data section.

### Starting materials

The starting material was a synthetic equivalent of the MORB, prepared by mixing high-purity powdered oxides and carbonates. The starting powders were pre-dried, mixed in agate mortar and decarbonated in an iron-saturated Pt crucible in a box furnace by increasing temperature from 650 to 1000°C over 7 h, then melted at 1550°C for 25 min and rapidly quenched.

### High-pressure, high-temperature experiment

LVP experiments were performed with an end-loaded piston cylinder press and a GY420 cubic press at Center for High Pressure Science & Technology Advanced Research (HPSTAR) in Beijing. Cell pressures were calibrated in Xu *et al*. [49]. For cubic-press experiments, the newly developed rapid-cooling assembly was utilized [50]. In this assembly, the conventional graphite furnace is replaced by a thin central graphite rod, which significantly increases the cooling rate after power shut-off. This design allows high-pressure melts to be quenched into homogeneous silicate glass without quenched mineral grains. This is essential to enable accurate measurements of volatile compound concentrations. Starting material powders were sealed into the welded Pt capsules or folded Re foil capsules in oxygen-rich conditions imposed by adding PtO_2_ powder. Notably, no PtO_2_ is present in the samples after the experiments, so the real *f*O_2_ in the experiments is more reduced than the *f*O_2_ of the Pt–PtO_2_ buffer. High-temperature, 1-atm experiment reported in Table 1 was performed in a box furnace, the temperature was calibrated using the melting point of gold.

### Analytical techniques and procedures

Room temperature Mössbauer spectroscopic analysis was performed in a transmission mode on a constant acceleration Mössbauer spectrometer with a nominal 370 MBq ^57^Co source in a 12 μm Rh matrix, at the Bayerisches Geoinstitut, Germany.

The chemical composition of run products was analysed using a JEOL JXA-8230 Electron Microprobe at the Testing Center of Shandong Bureau of China Metallurgical Geology Bureau. Natural and synthetic standards were used for calibration. Oxygen contents were measured using an LDE1 (layered dispersion element 1) analysing crystal and calibrated against the nominal oxygen contents of stoichiometric mineral standards.

The CO_2_ and H_2_O concentrations were measured using Cameca NanoSIMS 50 L instruments at the Institute of Geology and Geophysics, Chinese Academy of Sciences and the Guangzhou Institution of Geochemistry, Chinese Academy of Sciences. Measurements were calibrated against basaltic glass standards with known volatile contents determined by Fourier-transform infrared (FTIR) spectroscopy, and background levels were monitored using nominally dry reference materials.

Raman spectra of run product glasses were collected at HPSTAR Beijing using a confocal Renishaw inVia Raman spectrometer with 532 nm excitation, 1 cm^−1^ spectral resolution and calibration against the 520 cm^−1^ Si standard.

### Molecular dynamics calculations

*Ab initio* MD simulations were performed using the Vienna *ab initio* simulation package [51] to sample representative MgSiO*_x_* structures at high temperature. Single point density functional theory calculations were used to generate reference energies, forces and charge densities. Bader charge analyses were conducted to quantify the atomic charges. A high-precision DNN potential for MgSiO*_x_* (*x* ≥ 3) was developed to accurately reproduce the structural and dynamical properties of crystalline orthoenstatite and liquid MgSiO_3_ at high pressures and temperatures.

## Supplementary Material

nwag539_Supplemental_Files

## Data Availability

The raw data for molecular dynamics simulations, deep neural network potential training and Bader charge analysis have been publicly released on the GitHub repository at https://github.com/NWPU-Niu-Group/Excess-Oxygen-in-Magma. VASP can be acquired from VASP Software GmbH at https://www.vasp.at/. LAMMPS and DeePMD-kit are free and opensource codes available at https://www.lammps.org/ and https://deepmodeling.com/, respectively.
