# Supplementary material for: Magma as a carrier of non-stoichiometric oxygen linking Earth’s interior and atmosphere
Source: Natl Sci Rev. 2026 Aug 25;13(18):nwag539. doi: 10.1093/nsr/nwag539 (PMC13613983; doi:10.1093/nsr/nwag539)
Supplement: Supplementary file 1 [file nwag539_supplemental_files.zip › XuEtAl_Suppl_0820.pdf]

Supplementary Information for

Magma as a carrier of non-stoichiometric oxygen linking Earth's interior and atmosphere

Yongjiang Xu[1,2], Haiyang Niu[3], Wim van Westrenen[2,4], Ruiheng Liu[3], Peiyan Wu[1,2], Ho-Kwang Mao[1,5], Yanhao Lin[1,2*]

[1]Center for High Pressure Science and Technology Advanced Research, Beijing 100193, People's Republic of China
[2]Lin Earth and Planetary Laboratory, Center for High Pressure Science and Technology Advanced Research, Beijing 100193, People's Republic of China
[3]State Key Laboratory of Solidification Processing, International Centre for Materials Discovery, School of Materials Science and Engineering, Northwestern Polytechnical University, Xi'an 710072, People's Republic of China
[4]Department of Earth Sciences, Faculty of Science, Vrije Universiteit Amsterdam, De Boelelaan 1085, 1081 HV Amsterdam, The Netherlands
[5]Shanghai Advanced Research in Physical Sciences, Shanghai 201203, People's Republic of China

*Corresponding author. Email: yanhao.lin@hpstar.ac.cn

This file includes:

Methods

Starting materials

High-pressure, high-temperature experiments

Analytical techniques and procedures

Mössbauer spectroscopic analysis

Electron microprobe analysis (EMPA)

Secondary ion mass spectrometry (SIMS)

Raman Spectroscopy

Molecular dynamics calculations

Ab initio molecular dynamics

Density functional theory reference calculations

Bader charge analysis

Deep neural network potential development

Figures S1 to S8

Tables S1 to S4

Methods

Starting materials

Starting material was a synthetic mid-ocean ridge basalt (MORB). A synthetic equivalent of the mid ocean ridge basalt (MORB) composition from Melson *et al.* [1] was prepared by mixing high purity (99.5-99.99%, Alfa Aesar) powdered oxides including SiO_2, TiO_2, Al_2O_3, Fe_2O_3, MgO, and carbonates including $CaCO_3$, Na_2CO_3, and K_2CO_3. The oxides SiO_2, TiO_2, Al_2O_3, and MgO were fired overnight at 1100 °C, and Fe_2O_3, $CaCO_3$, Na_2CO_3 and K_2CO_3 were stored at 110 °C overnight before use. After mixing all the oxides with appropriate weights under ethanol in an agate mortar for 1 h, mixtures were dried in a fume hood and decarbonated in an iron-saturated Pt crucible in a box furnace by increasing temperature from 650 to 1000 °C over 7 h. Resulting mixtures were melted for 25 min at 1550 °C to ensure chemical homogenisation. The melts were quenched rapidly by immersing the bottom of Pt crucible in water. A small part of the resulting glass was mounted in epoxy, ground, polished and carbon-coated to analyze its chemical composition. Most of remaining glass was crushed separately into powder in a ball mill for 90 minutes, and then stored at 110 °C for at least 24 hours prior to use. Because the valence proportions of multivalent iron were measured in the MORB starting material, described below, by Mössbauer spectroscopic analysis, its EO could be determined very precisely at 0.0 ± 0.1 wt.% (Table 1, Fig. 1).

High-pressure, high-temperature experiments

Large-volume high-pressure (LVP) experiments were conducted in welded Pt capsules or folded Re foil capsules (0.7mm ID, 1mm OD, 1 mm length) in oxygen-rich conditions imposed by adding PtO_2 powder (Alfa Aesar, purity of 99.95%; $\log fO_2 \approx 0$) to the starting material in the Pt and Re capsules, respectively. Twenty-one high pressure and temperature experiments (Table 1) were conducted under nominally dry conditions following the procedures described in Lin *et al.* [2]. Representative backscattered electron images of typical run products of LVP experiments with the MORB composition are shown in Fig. S6a. All run products contain glass (quenched magma), those that were performed at sub-liquidus conditions in addition contain one or more mineral phases. Both qualitative element mapping (Fig. S6b) and quantitative EPMA analyses show that oxygen is distributed homogeneously in the quenched

glasses. As detailed below it is impossible to prevent water (and CO_2) incorporation into the melts during these LVP experiments. Chlorine was identified as an impurity in the PtO_2 powder, and its effect on overall electroneutrality of the silicate melts was also taken into account.

Sample pressures and temperatures in the LVP experiments ranged between 0.5-7 GPa and 1200-1800 °C, respectively. Experiments at 0.5-3 and 3.4-7 GPa were performed with an end-loaded piston cylinder press and a GY420 cubic press at Center for High Pressure Science & Technology Advanced Research (HPSTAR) in Beijing [3,4], respectively. The piston cylinder press experiments [5] used a pressure cell consisting of a 13 or 19 mm diameter concentric outer talc sleeve and inner Pyrex glass, a graphite furnace and crushable MgO spacers. These experiments were first pressurized to 300 psi at room temperature, after which temperature (100 K/min ramp) and pressure were raised simultaneously to their target values. Dwell times at target temperatures were 5 minutes. Temperatures were monitored using a $Pt_{70}Rh_{30}$-$Pt_{94}Rh_6$ (type B) thermocouple and a Eurotherm 2404 programmable controller. The temperature readings were adjusted upwards by 10 °C to take account of the distance between thermocouple tip and capsule [6], and a pressure correction of 3% was applied based on pressure calibrations using the approach described in van Kan *et al.* [7] and McDade *et al.* [8]. All the experiments were quenched by cutting power to the furnace, with temperatures dropping below 100 °C in < 7 s.

Cubic press experiments were performed in a six-anvil cubic press with a maximum force of 14 MN on every WC anvil [3,4]. To create high pressure, six WC anvils with a square-shaped truncation driven from three perpendicular dimensions by a computer-controlled hydraulic system pressurize a central pyrophyllite cubic block (38.5 x 38.5 x 38.5 mm^3), which serves as both pressure medium and gasket material. In the experiments mentioned above, the cell pressures were calibrated by observing the phase transitions of Bi (I-II transition at 2.55 GPa and III-V at 7.6 GPa), Cd_3P_2 (I-II transition at 4 GPa) and Ba (I-II transition at 5.55 GPa) [9-14]. The phase transitions were detected using the resistance measurement method described in Xu *et al.* [4]. Temperature was monitored using a $W_{97}Re_3$-$W_{75}Re_{25}$ (type D) thermocouple and Eurotherm 2404 programmable controller. The Pt or Re capsule was located at the hotspot of the assembly, within 1 mm of the thermocouple conjunction point to minimize thermal gradients and achieve sample temperatures within 5 °C of the thermocouple reading [6]. Experiments were pressurized cold to target values, and then

heated while maintaining pressure. Experiment duration was 2 minutes. After completion of an experiment, runs were quenched by cutting power to the heater and the temperature typically dropped below the glass transition in < 2 s [3]. Pressure effects on the thermoelectromotive force of the thermocouples was not considered in either the piston cylinder or the cubic press experiments.

The single high-temperature, 1-atm experiment reported in Table 1 was performed in a box furnace, using MORB glass starting material mixed with PtO_2 powder (Alfa Aesar, purity of 99.95%) in a welded Pt capsule (0.7mm ID, 1mm OD, 2 mm length). The box furnace temperature was calibrated before the experiments using the melting point of gold, and the experiment ran for 5 mins above the liquidus. The temperature was monitored with a $Pt_{70}Rh_{30}$-$Pt_{94}Rh_6$ (type B) thermocouple, and the sample was quenched in water.

Analytical techniques and procedures

Mössbauer spectroscopic analysis

To determine the proportion of trivalent iron in our MORB starting material, a glass shard of the synthetic glass with approximate dimensions 1100 x 600 x 500 μm^3 was used, corresponding to a Mössbauer thickness of roughly 10 mg Fe/cm^2. A Mössbauer spectrum was recorded at room temperature in transmission mode on a constant acceleration Mössbauer spectrometer with a nominal 370 MBq ^{57}Co source in a 12 μm Rh matrix, at the Bayerisches Geoinstitut, Germany. Gamma rays were collimated using a 500 μm hole in lead foil. The velocity scale was calibrated relative to 25 μm thick α-Fe foil; line widths of 0.36 mm/s for the outer lines of α-Fe were obtained at room temperature. The spectrum was collected for 7 days and was fit using the full transmission integral in the program MossA [15]. The spectrum is typical for basaltic glass and was fit according to established models [16] to two doublets with pseudo-Voigt line shape (Fig. S7). The value of $Fe^{3+}/\Sigma Fe$ was determined from relative areas to be 0.70 ± 0.03 (Fig. S7). Hyperfine parameters are listed in Table S1.

Electron microprobe analysis (EMPA)

Experimental run products were mounted in epoxy, polished and carbon-coated for back-scattered electron (BSE) imagery used to assess the texture and mineralogy, and for quantitative compositional measurements using electron microprobe analysis

(EMPA). The chemical composition of run product phases (quenched glass) was determined using a JEOL JXA-8230 Electron Microprobe at the Testing Center of Shandong Bureau of China Metallurgical Geology Bureau. Two analytical protocols were used. Measurements of the concentrations of Si, Ti, Al, Fe, Mg, Ca, Na, K, Cl, Pt, Re and O used an accelerating voltage of 15 kV and a beam current of 10 nA. Beam size of 10-20 μm diameters were used for the homogeneous glass. Compositions of glass were determined with average values of 2-10 analysis points. Analyses were calibrated daily against primary standards comprising natural samples of forsterite for Mg, jadeite for Na, sanidine for K, and fayalite for Fe, tugtupite for Cl, metal Pt and Re for Pt and Re, respectively, and synthetic oxides rutile for Ti and Smithsonian basaltic glass standard VG-2 for Si, Al, and Ca. O analyses were calibrated daily by comparing the nominal O contents of stoichiometric mineral standards of magnetite (O = 27.67 wt.%), rhodonite (37.81 wt.%), olivine (43.89 wt.%), pyrope (44.30 wt.%) and obsidian (48.63 wt.%), with the directly measured O contents by using an LDE1 analyzing crystal. Sets of O calibration data are plotted in Fig. S8. Peak areas were converted to concentrations using standard values. Peak count times were 20 seconds and background count times were 10 seconds. A summary of individual EPMA analyses for starting materials and experimental run products used in this study is provided in Table S2.

Secondary ion mass spectrometry (SIMS)

The CO_2 and H_2O equivalent concentrations in starting materials and run products were measured using nanoSIMS instruments at the Institute of Geology and Geophysics, Chinese Academy of Sciences (IGGCAS) and Guangzhou Institution of Geochemistry, Chinese Academy of Sciences (GIGCAS). At IGGCAS, samples were remounted in alloy mounts, and then polished by a diamond polishing medium (0.5 μm polycrystalline diamond suspension). The mount was dried in an oven for several days before undergoing coating with platinum for nanoSIMS analysis using a Cameca NanoSIMS 50L. The mounted samples were stored in the airlock of the NanoSIMS, baked for 72 hours, and then loaded into the analysis chamber. Four basaltic glass standards (IND-G1 with 0.5 wt.% H_2O and 97 ppm CO_2, IND-G2 with 0.5 wt.% H_2O and 389 ppm CO_2, EPR-G3 with 0.22 wt.% H_2O and 269 ppm CO_2, FJ-G2 with 0.23 wt.% H_2O and 307 ppm CO_2) were used to calibrate the CO_2 and water equivalent contents. The CO_2 and H_2O contents in these standards were previously determined by

Fourier transform infrared (FTIR) spectroscopy [17]. Detection limits were determined by analysis of nominally dry San Carlos olivine, which has H_2O content < 1 ppm [18] and C content < 2 ppm [19]. During sample analysis, a liquid nitrogen cold trap was used, which further reduced the vacuum of the analysis chamber down to 3~4 × 10^{-10} torr [20,21]. Analyses are made using a focused Cs^+ primary beam with a current of ~500 pA and an accelerating voltage of 16 kV. The size of the analytical area was as small as 3×3 - 5×5 μm^2. The real time image (RTI) was used to select clean sample areas with no cracks or craters to minimise surface contamination [18,22,23]. During each cycle, ^{1}H, ^{12}C and ^{18}O were collected simultaneously. $^{1}H/^{18}O$ ratios and $^{12}C/^{18}O$ ratios were calibrated to yield the CO_2 and H_2O contents, as illustrated in Table S3. At GIGCAS, measurements of CO_2 and H_2O contents were conducted using the Cameca NanoSIMS 50L following the method described in Yang *et al.* [24]. A primary beam of Cs^+ was used to raster the target sample area and negative secondary ions $^{12}C^-$, $^{16}O^{1}H^-$, and $^{30}Si^-$, were collected simultaneously with electron multipliers. The nuclear magnetic resonance probe was activated along with the Hall probe to correct potential magnetic field drift and ensure a stable magnetic field during the analysis. The electron gun was applied to compensate potential charging effects. Before each analysis, a primary beam of 2 nA was used to sputter the target area of 30 μm × 30 μm for 4 minutes, after which a primary beam of 0.5 nA was used to raster the central 20 μm × 20 μm. Only signal from the central 6.7 μm × 6.7 μm area was recorded with electronic blanking applied. Fifty cycles of data with 0.54 second for each cycle were collected. The data were corrected for EM deadtime (44 ns). ^{30}Si-normalized secondary ion ratios of basaltic glass standards EPR-G3, IND-G1, IND-G2, FJ-G2, MA42 [17] and ALV519-4-1 [25] were used to construct calibration curves through the origin and derive sensitivity factors to calibrate CO_2, and H_2O contents of unknown glass samples. The rhyolitic glass standard MRN-G1 [17] was used to calibrate H_2O content of glass samples with high SiO_2 content. The glass standard ALV519-4-1 [25] was analyzed for each session to keep track of instrumental drift and derive corrected sensitivity factors as adopted by Kumamoto *et al.* [26]. The background levels of CO_2 and H_2O were monitored using nominally dry Suprasil 3002 glass. The detailed analytical data used to calibrate and calculate the CO_2 and H_2O contents are shown in Table S4.

NanoSIMS analyses indicate that the hydrogen content of the MORB glass is 380 ±38 ppm H_2O equivalent, and the CO_2 equivalent content is 26 ±7 ppm, consistent with their low solubilities at 1 atm pressure [27]. The resulting bulk water equivalent

and CO_2 contents obtained at both GIGCAS and IGGCAS are shown in Table 1, and Tables S3 and S4.

Raman Spectroscopy

Raman spectra of run product glasses were collected using an InSpect confocal Raman microscope equipped with a Renishaw inVia Raman spectrometer, located at HPSTAR Beijing. The excitation wavelength was 532 nm (green light) from a 50 mW laser. A Peltier-cooled CCD detector, and a grating of 2400 grooves/mm were used, resulting in a spectral resolution of 1 cm^{-1}. Spectra were collected in confocal setting with a 20× objective (spatial resolution of less than 2 μm). Exposure time of 10s and 20 accumulations were set for each measurement. Raman spectra were calibrated by a silicon wafer at 520 cm^{-1} Raman shift, and the calibration of the instrument was checked at the beginning of each analytical session.

Molecular dynamics calculations

Ab initio molecular dynamics

Ab initio molecular dynamics (AIMD) simulations were performed using the Vienna *ab initio* simulation package (VASP) [28]. The PBEsol exchange-correlation functional [29] was used to maintain consistency with the previously developed $MgSiO_3$ potential [30,31]. Electronic wavefunctions were represented by a plane-wave basis with a cutoff energy of 400 eV, and electronic energies were converged to 10^{-6} eV. The Monkhorst-Pack k-point mesh was set to 1×1×1. The AIMD simulations were conducted in the canonical (NVT) ensemble using a Nose-Hoover thermostat [32] and a timestep of 1 fs. Periodic boundary conditions were applied throughout.

Density functional theory reference calculations

Single point density functional theory (DFT) calculations used to generate reference energies and forces for training the machine-learning potential were carried out using the same computational framework as the AIMD simulations, with two modifications: the plane-wave cutoff energy was increased to 800 eV and the Monkhorst-Pack *k*-point mesh was expanded to 2 × 2 × 2. These calculations provided the reference dataset for the construction and validation of the deep neural network (DNN) potential.

Bader charge analysis

Bader charge analyses were conducted to quantify the atomic charges in the system by using the charge densities obtained from DFT single point calculations (CHGCAR file). The charge density was partitioned into atom-associated Bader volumes to determine atomic charges. The analyses employed the grid-based algorithm of Henkelman *et al.* [33]. Three representative structures containing (Mg, Si, O) = (46, 46, 138), (46, 46, 143), (49, 49 ,154) were examined. All computational convergence and parameters were identical to those used for the corresponding single point DFT calculations.

Deep neural network potential development

A high-precision deep neural-network (DNN) potential for $MgSiO_x$ ($x \geqslant 3$) was developed using the DeePMD-Kit package [34]. The potential was constructed by extending a previously validated $MgSiO_3$ model [30,31], which accurately reproduces both the structural and dynamical properties of crystalline orthoenstatite and liquid $MgSiO_3$ over a wide range of pressures and temperatures. To capture the oxygen-rich compositions relevant to this study, the training set was expanded to include configurations of $MgSiO_x$ liquids ($x \geqslant 3$), pure oxygen and mixed $MgSiO_x$-O_2 systems, which help to determine the excess oxygen contents under different conditions.

The new dataset was generated through an iterative procedure that combines AIMD simulations under various temperature and pressure conditions, as well as long timescale machine learning molecular dynamics (MLMD) simulations spanning oxygen contents equivalent to a range from $x = 3$ to ~3.5.

For the AIMD simulations, the initial configurations were constructed as coexisting systems of $MgSiO_x$-O_2 and oxygen. The atomic compositions of the three simulations are ($Mg_0Si_0O_{184}$), ($Mg_{32}Si_{32}O_{100}$), and ($Mg_{20}Si_{20}O_{144}$), respectively. All three AIMD simulations were performed under the NVT ensemble. For the liquid oxygen simulation, the pressure was approximately 9 GPa, the temperature was around 1900 °C. For the $MgSiO_x$-O_2 gas system, the initial structure was built from ($Mg_{32}Si_{32}O_{96}$) with a vacuum slab introduced on the surface. An AIMD run at about 1400 °C was first carried out to obtain the vacuum-interface configurations of $MgSiO_3$. Meanwhile, two O_2 molecules were placed in the vacuum region to approximate the O_2 gas, and the simulation temperature ranged approximately between 1350 °C and

1550 °C. For the $MgSiO_x$-liquid O_2 system, the simulation pressure was about 6.5 GPa, the temperature was approximately 1900 °C. The above describes the AIMD simulation stage, after which the generated configurations were used in the subsequent active iteration process. For the MLMD simulations, we performed iterations within the temperature and pressure ranges where the excess oxygen content was determined. The four models described in the AIMD section were employed in the MLMD iterations.

The final training dataset comprises 74,372 structures, representing the relevant high-dimensional atomic environments, including 33,694 configurations newly added during the iterative sampling procedure, while the rest is taken from the literature which contains the solid and liquid configurations of $MgSiO_3$ [30,31]. The new dataset were divided into training and test subsets in a 9:1 ratio.

Comprehensive coverage of the configurational space was confirmed through principal component analysis (PCA) of smooth overlap of atomic positions (SOAP) [35] descriptors (Fig. S3). This analysis ensured that all relevant configurations, including $MgSiO_x$, solid $MgSiO_3$, pure oxygen, and $MgSiO_x$-liquid oxygen coexisting configurations, are sampled, thereby demonstrating the robust descriptive capability of the DNN potential.

The neural network architecture included embedding layers of size [25, 50, 100] and fitting layers of [240, 240, 240]. A smoothly decaying cutoff radius from 5.8 Å to 6.0 Å was adopted. The learning rate was decayed from 1.0×10^{-3} to 3.5×10^{-8}. In the loss function, the weight coefficients for energies and forces were varied from 0.02 to 2 and 1000 to 1, respectively. The $MgSiO_x$ dataset was divided into training and test subsets in a 9:1 ratio. Model accuracy was assessed by comparison against DFT reference data, yielding root-mean-square errors of 6.41 meV/atom for energies and 0.312 eV/Å for forces on the test set.

Regarding the choice of oxygen phase, we clarify that liquid oxygen was not used to represent the oxygen phase under all conditions. According to the oxygen phase diagram, oxygen is gaseous at 1 bar under the relevant temperature conditions. Therefore, for the 1 bar case, we explicitly placed O_2 molecules in the vacuum region above the $MgSiO_3$ surface and performed NVT simulations to maintain the gas region and evaluate the equilibrium excess oxygen content at 1 bar. These gas-phase O_2-containing configurations were also included in the training dataset to ensure that the DNN potential covered this part of configuration space. In contrast, at pressures of 3–9 GPa, oxygen is in a dense fluid state rather than a gas. Therefore, for these high-

pressure conditions, we used $MgSiO_x$-liquid O_2 coexistence models under the NPT ensemble. This choice is consistent with the oxygen phase diagram and provides the appropriate oxygen reservoir at high pressure. Thus, the use of liquid/fluid oxygen does not introduce an artificial bias into the high-pressure results; instead, it reflects the physically relevant oxygen phase under those conditions.

References

1. Melson WG, Vallier TL, Wright TL *et al*. Chemical diversity of abyssal volcanic glass erupted along Pacific, Atlantic, and Indian Ocean sea-floor spreading centers. *Geophys Monogr Ser* 1976; **19**: 351-67.
2. Lin Y, Tronche EJ, Steenstra ES *et al*. Experimental constraints on the solidification of a nominally dry lunar magma ocean. *Earth Planet Sci Lett* 2017; **471**: 104-16.
3. Wu P, Xu Y, Lin Y. A novel rapid cooling assembly design in a high-pressure cubic press apparatus. *Matter Radiat Extrem* 2024; **9**: 025401.
4. Xu Y, Wu P, Shang S *et al*. Pressure calibrations of high-pressure large-volume presses at HPSTAR. *Matter Radiat Extrem* 2026; **11**: 017803.
5. Boyd F and England J. Apparatus for phase-equilibrium measurements at pressures up to 50 kilobars and temperatures up to 1750 °C. *J Geophys Res* 1960; **65**: 741-8.
6. Watson E, Wark D, Price J *et al*. Mapping the thermal structure of solid-media pressure assemblies. *Contrib Mineral Petrol* 2002; **142**: 640-52.
7. van Kan Parker M, Mason PR, van Westrenen W. Experimental study of trace element partitioning between lunar orthopyroxene and anhydrous silicate melt: effects of lithium and iron. *Chem Geol* 2011; **285**: 1-14.
8. McDade P, Wood BJ, Van Westrenen W *et al*. Pressure corrections for a selection of piston-cylinder cell assemblies. *Mineral Mag* 2002; **66**: 1021-8.
9. Heydemann PL. The Bi I-II transition pressure measured with a dead-weight piston gauge. *J Appl Phys* 1967; **38**: 2640-4.
10. Heydemann PL. Erratum: The Bi I-II transition pressure measured with a dead-weight piston gauge. *J Appl Phys* 1967; **38**: 3424.
11. Decker DL, Bassett WA, Merrill L *et al*. High-pressure calibration: a critical review. *J Phys Chem Ref Data* 1972; **1**: 773-836.
12. Ono S. High-pressure phase transition of bismuth. *High Press Res* 2018; **38**: 414-21.
13. Huang M, Peng F, Guan S *et al*. Powder conductor for pressure calibration applied to large-volume press under high pressure. *Rev Sci Instrum* 2021; **92**: 073903.

14. Andersson G, Sundqvist B, Bäckström G. A high-pressure cell for electrical resistance measurements at hydrostatic pressures up to 8 GPa: results for Bi, Ba, Ni, and Si. *J Appl Phys* 1989; **65**: 3943-50.

15. Prescher C, McCammon C, Dubrovinsky L. MossA: a program for analyzing energy-domain Mössbauer spectra from conventional and synchrotron sources. *J Appl Crystallogr* 2012; **45**: 329-31.

16. Partzsch GM, Lattard D, McCammon C. Mössbauer spectroscopic determination of Fe^{3+}/Fe^{2+} in synthetic basaltic glass: a test of empirical fO_2 equations under superliquidus and subliquidus conditions. *Contrib Mineral Petrol* 2004; **147**: 565-80.

17. Shimizu K, Ushikubo T, Murai T *et al*. In situ analyses of hydrogen and sulfur isotope ratios in basaltic glass using SIMS. *Geochem J* 2019; **53**: 195-207.

18. Li R, Hao J, Hu S *et al*. High-spatial-resolution measurement of water content in olivine using NanoSIMS 50L. *At Spectrosc* 2022; **43**: 77-83.

19. Mathez EA and Delaney JR. The nature and distribution of carbon in submarine basalts and peridotite nodules. *Earth Planet Sci Lett* 1981; **56**: 217-32.

20. Hao J, Hu S, Li R *et al*. High precision and resolution chlorine isotopic analysis of apatite using NanoSIMS. *At Spectrosc* 2022; **43**: 321-28.

21. Hao J, Yang W, Hu S *et al*. Submicron spatial resolution Pb-Pb and U-Pb dating by using a NanoSIMS equipped with the new radio-frequency ion source. *J Anal At Spectrom* 2021; **36**: 1625-33.

22. Hao J, Zhang L, Yang W *et al*. NanoSIMS sulfur isotopic analysis at 100 nm scale by imaging technique. *Front Chem* 2023; **11**: 1120092.

23. Hao J, Yang W, Huang W *et al*. NanoSIMS measurements of sub-micrometer particles using the local thresholding technique. *Surf Interface Anal* 2020; **52**: 234-39.

24. Yang YN, Du Z, Lu W *et al*. NanoSIMS analysis of water content in bridgmanite at the micron scale: an experimental approach to probe water in Earth's deep mantle. *Front Chem* 2023; **11**: 1166593.

25. Hauri EH, Weinreich T, Saal AE *et al*. High pre-eruptive water contents preserved in lunar melt inclusions. *Science* 2011; **333**: 213-15.

26. Kumamoto KM, Warren JM, Hauri EH. New SIMS reference materials for measuring water in upper mantle minerals. *Am Mineral* 2017; **102**: 537-47.

27. Lichtenberg T, Bower DJ, Hammond M *et al*. Vertically resolved magma ocean-

protoatmosphere evolution: H_2, H_2O, CO_2, CH_4, CO, O_2, and N_2 as primary absorbers. *J Geophys Res Planets* 2021; **126**: e2020JE006711.

28. Kresse G and Furthmüller J. Efficiency of ab initio total energy calculations for metals and semiconductors using a plane-wave basis set. *Comput Mater Sci* 1996; **6**: 15-50.
29. Perdew JP, Ruzsinszky A, Csonka GI *et al*. Restoring the density-gradient expansion for exchange in solids and surfaces. *Phys Rev Lett* 2008; **100**: 136406.
30. Deng J, Niu H, Hu J *et al*. Melting of $MgSiO_3$ determined by machine learning potentials. *Phys Rev B* 2023; **107**: 064103.
31. Zhang S, Hu J, Sun X *et al*. Structural heterogeneity of $MgSiO_3$ liquid and its connection with dynamical properties. *Phys Rev Lett* 2025; **134**: 204101.
32. Hoover WG. Canonical dynamics: equilibrium phase-space distributions. *Phys Rev A* 1985; **31**: 1695.
33. Henkelman G, Arnaldsson A, Jonsson H. A fast and robust algorithm for Bader decomposition of charge density. *Comput Mater Sci* 2006; **36**: 354-60.
34. Wang H, Zhang L, Han J. DeePMD-kit: a deep learning package for many-body potential energy representation and molecular dynamics. *Comput Phys Commun* 2018; **228**: 178-84.
35. Bartók AP, Kondor R, Csányi G. On representing chemical environments. *Phys Rev B* 2013; **87**: 184115.

Fig. S1. Excess oxygen in silicate glass as a function of glass concentrations of (a) CO_2 and (b) H_2O. The dotted line indicates the near-zero excess oxygen value of the starting materials.

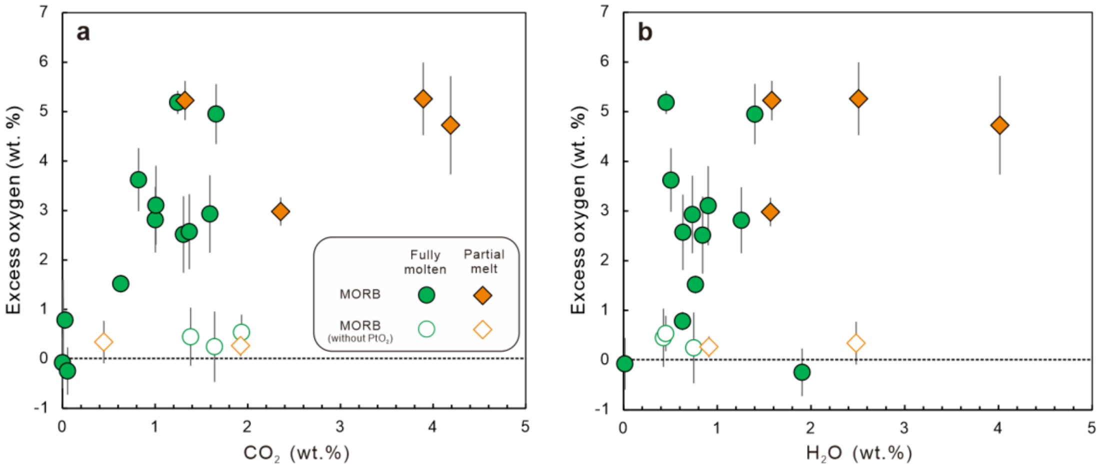

Fig. S2. Excess oxygen in silicate glass plotted against the concentrations of elemental abundances of (a) Si, (b) Al, (c) Fe, (d) Mg, (e) Ca and (f) Na.

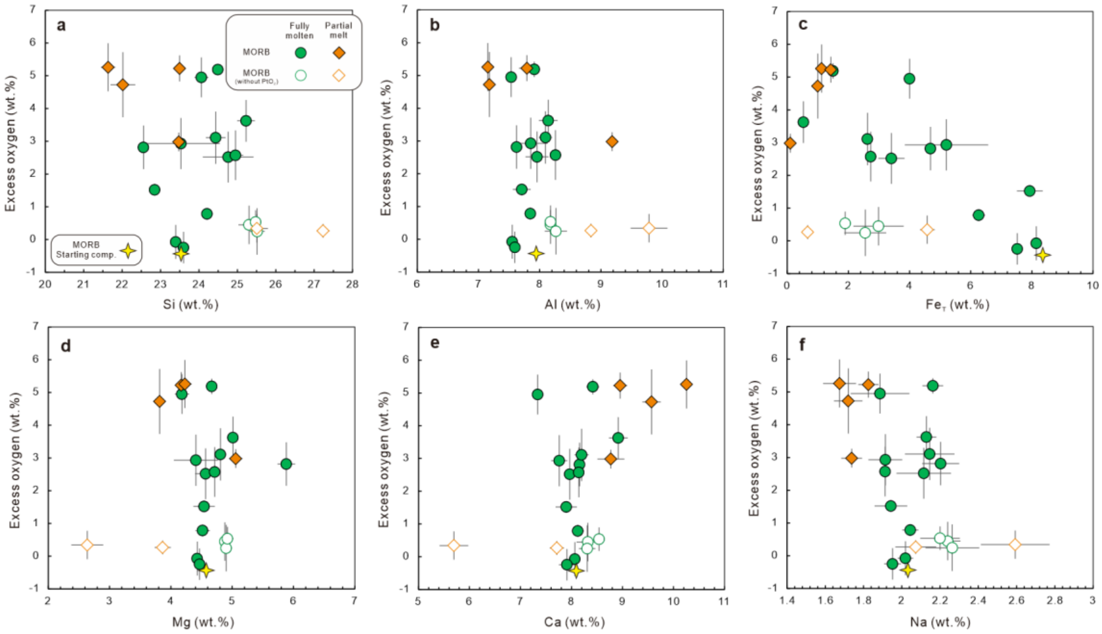

Fig. S3. Principal component analysis map of the training set configurations for the DNN and test set prediction errors. (**a)** The energy of each configuration is colored from red to blue. Typical structures of $MgSiO_x$, solid $MgSiO_3$, liquid oxygen and $MgSiO_x$-liquid oxygen coexisting configurations are shown, respectively. (**b)** and **(c)** Comparison of energies and forces predicted by the DNN potential with DFT, respectively.

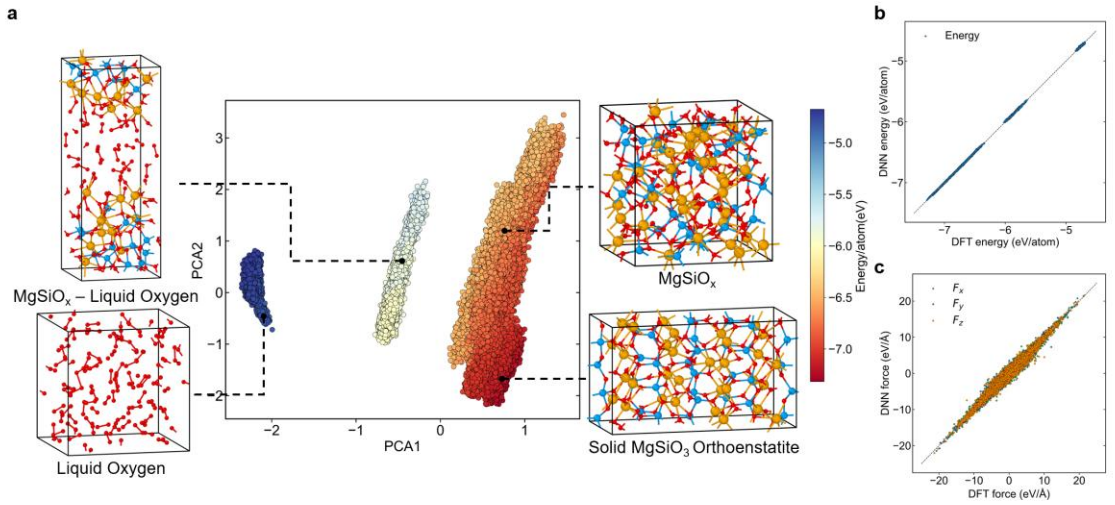

Fig. S4. Bader charge distributions of Mg, Si and O as a function of excess-oxygen content. The distributions demonstrate that Mg and Si maintain nearly constant charge states, whereas oxygen exhibits progressively broader and less negative charge values as oxygen content increases.

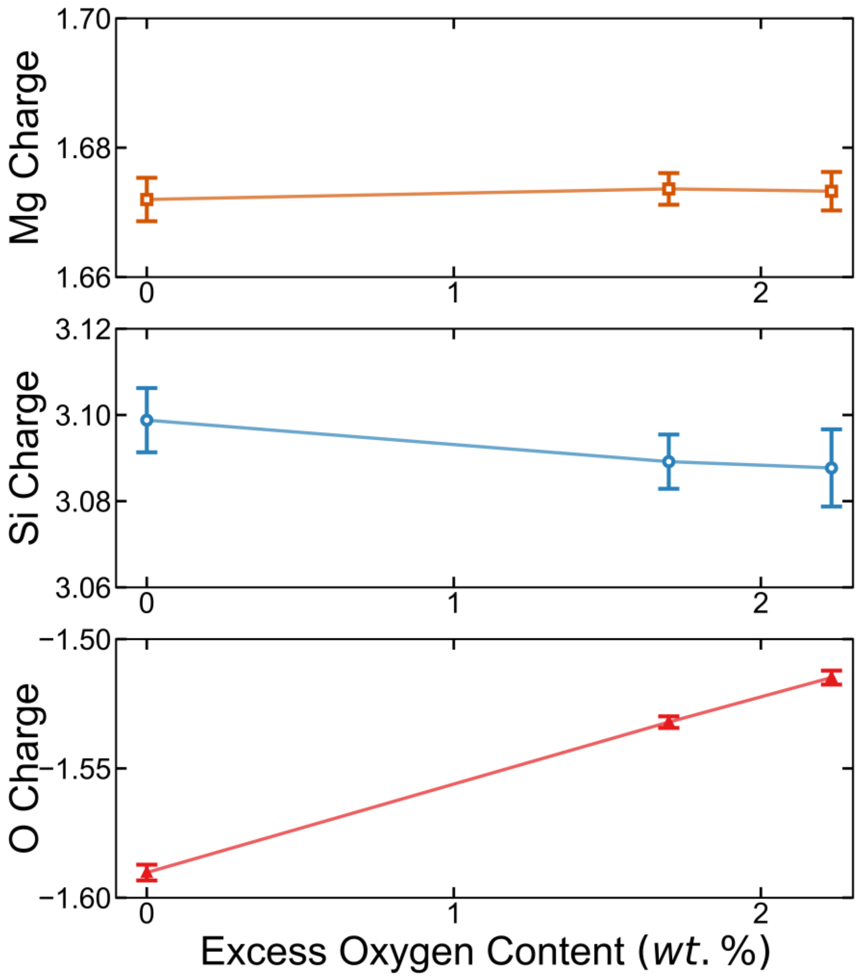

Fig. S5. Representative Raman spectra of starting materials and quenched glass in experiments using MORB in LVP, discussed in the main text. Note that a $(CO_3)^{2-}$ peak is present around 1100 cm^{-1} in samples LVP-7 and LVP-19, but not in LVP-2 or our MORB starting glass. This is consistent with the SIMS carbon measurements shown in Table 1.

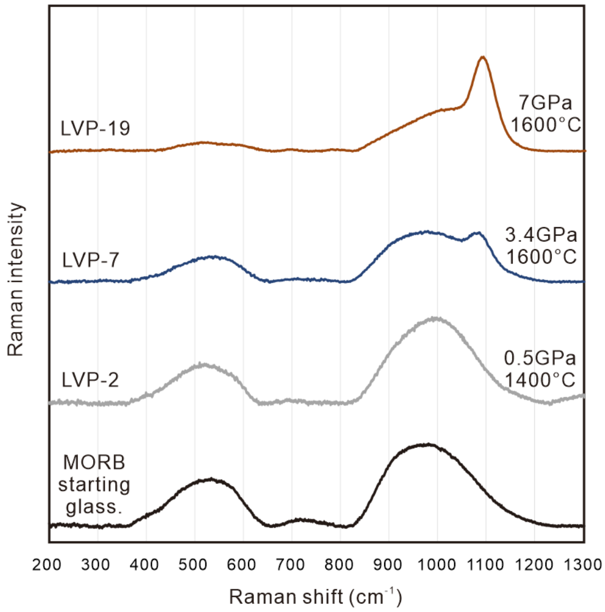

Fig. S6. Representative images of backscatter electron (BSE) (a) and oxygen qualitative element mapping (b) of the experimental run products at a pressure of 7 GPa. Qgl, quenched glass with crystal aggregates; Gl, homogenous glass.

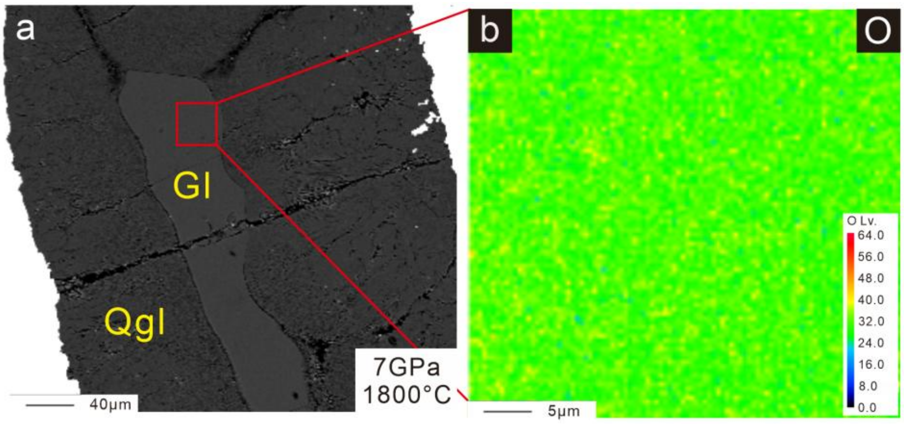

Fig. S7. Room temperature Mössbauer spectrum of MORB glass starting material fitted to two doublets with pseudo-Voigt line shape assigned to Fe^{2+} (blue) and Fe^{3+} (green). The fit residual (difference between experimental and calculated data) is shown above the spectrum.

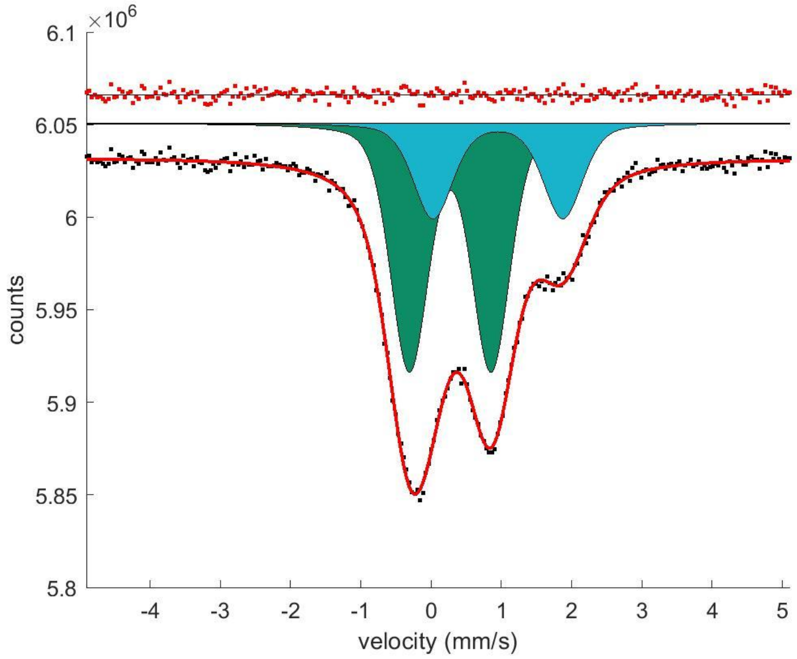

Fig. S8. Measured O on the calibration crystals against nominal O contents. The standards are magnetite (O wt.%, 27.67 wt.%), rhodonite (37.81 wt.%), olivine (43.89 wt.%), pyrope (44.30 wt.%) and obsidian (48.63 wt.%). Multiple symbols are shown for each standard, reflecting measurements performed during different analytical sessions. Dotted line shows best-fit linear calibration curve. Note that measured O contents in our run product glasses are between 43 and 50 wt.%

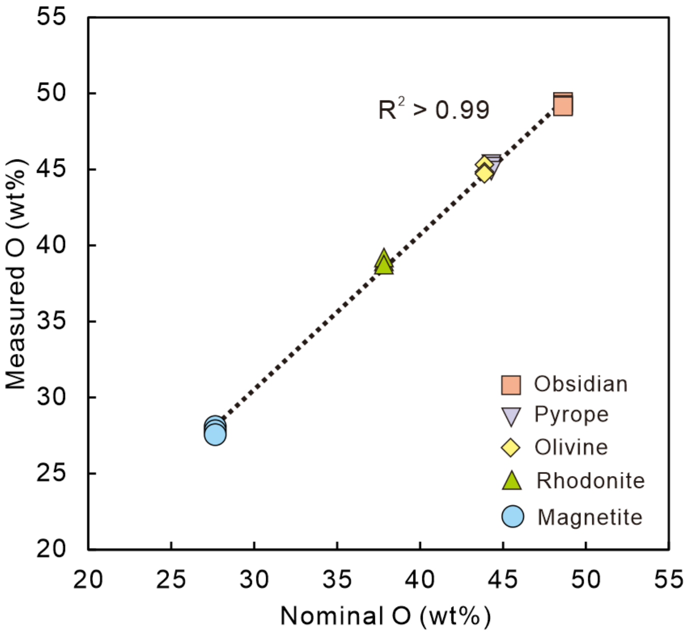

Table S1. Hyperfine parameters derived from room temperature spectrum of synthetic MORB glass starting material.

**Fe^{2+}**	
CS (mm/s)	0.95(2)
QS (mm/s)	1.85(3)
FWHM (mm/s)	0.67(5)
area (%)	30(3)
**Fe^{3+}**	
CS (mm/s)	0.27(1)
QS (mm/s)	1.16(1)
FWHM (mm/s)	0.64(2)
area (%)	70(3)

CS: center shift relative to α-Fe, QS: quadrupole splitting, FWHM: full width at half maximum, area: relative to total area; Values in brackets are uncertainties of the final digit.

Table S2. Summary of individual EPMA analysis for starting materials and experimental run products of this study (concentrations in wt.%).

Sample	Filename	Si	Ti	Al	Fe_T	Mg	Ca	Na	K	O	Re	Cl	Pt	Total
MORB glass starting comp.	MORB starting comp. 1	23.40	0.59	7.98	8.80	4.63	8.38	1.98	0.17	44.16				100.08
	MORB starting comp. 2	23.55	0.60	7.98	8.50	4.63	8.20	1.99	0.18	44.60				100.23
	MORB starting comp. 3	23.10	0.63	7.89	8.62	4.60	8.38	1.87	0.17	44.62				99.88
	MORB starting comp. 4	23.53	0.61	7.92	8.80	4.41	8.19	2.13	0.21	44.21				100.01
	MORB starting comp. 5	23.53	0.61	7.92	8.78	4.41	8.19	2.13	0.21	44.58				100.36
	MORB starting comp. 6	23.69	0.60	7.78	8.35	4.54	8.40	2.00	0.21	44.81				100.37
	MORB starting comp. 7	23.20	0.63	7.90	9.01	4.57	8.49	1.78	0.18	44.50				100.26
	MORB starting comp. 8	23.80	0.62	7.83	8.31	4.56	8.05	2.08	0.18	44.80				100.23
	MORB starting comp. 9	23.51	0.57	7.99	8.54	4.62	8.08	2.07	0.17	44.30				99.85
LVP-1	MORB+PtO2 1atm 1400 5min-1	23.31	0.61	7.53	8.26	4.44	8.09	1.97	0.25	44.07		0.63	0.00	99.17
	MORB+PtO2 1atm 1400 5min-2	23.43	0.57	7.58	8.05	4.47	7.99	2.04	0.17	43.59		0.60	0.00	98.48
	MORB+PtO2 1atm 1400 5min-3	23.45	0.56	7.53	8.13	4.38	8.13	2.04	0.17	44.15		0.63	0.00	99.16
LVP-2	MORB+PtO2 0.5Gpa 1400 5min-1	24.28	0.60	7.83	6.12	4.60	8.04	2.08	0.20	45.90		0.13	0.01	99.79
	MORB+PtO2 0.5Gpa 1400 5min-5	24.14	0.59	7.86	6.41	4.43	8.21	2.02	0.20	46.03		0.12	0.07	100.08
LVP-3	MORB+PtO2 0.5Gpa 1500 5min-2	23.67	0.55	7.57	7.46	4.45	8.08	1.93	0.18	45.43		0.14	0.00	99.45
	MORB+PtO2 0.5Gpa 1500 5min-3	23.46	0.63	7.53	7.50	4.51	7.88	2.00	0.21	45.72		0.16	0.07	99.66
	MORB+PtO2 0.5Gpa 1500 5min-5	23.67	0.55	7.68	7.62	4.45	7.77	1.93	0.23	45.48		0.14	0.00	99.51
LVP-4 (Cpx+Gl)	MORB nopt 1GPa 1200 3h-cpx2	22.91	0.47	3.93	6.93	8.87	12.48	0.51	0.02	43.89		0.01	0.09	100.11
	MORB nopt 1GPa 1200 3h-cpx3	23.24	0.40	3.94	6.54	9.05	12.10	0.47	0.00	43.49		0.00	0.12	99.36
	MORB nopt 1GPa 1200 3h-cpx4	23.38	0.37	4.08	5.45	8.55	13.37	0.53	0.04	44.29		0.01	0.10	100.16
	MORB nopt 1GPa 1200 3h-cpx5	23.32	0.47	4.01	6.78	8.99	12.60	0.53	0.04	44.64		0.00	0.00	101.38
	MORB nopt 1GPa 1200 3h-gl-1	25.50	0.72	9.48	4.64	2.95	6.16	2.40	0.27	47.50		0.00	0.00	99.63
	MORB nopt 1GPa 1200 3h-gl-2	25.65	0.64	9.50	4.34	2.81	5.77	2.64	0.33	48.11		0.01	0.04	99.83
	MORB nopt 1GPa 1200 3h-gl-3	25.99	0.68	10.21	4.37	2.27	5.29	2.90	0.38	48.23		0.03	0.05	100.38
	MORB nopt 1GPa 1200 3h-gl-4	25.25	0.74	10.07	4.59	2.54	5.61	2.61	0.32	48.52		0.02	0.00	100.26
	MORB nopt 1GPa 1200 3h-gl-5	25.20	0.87	9.65	5.01	2.81	5.69	2.43	0.34	48.22		0.00	0.00	100.23
	MORB nopt 1GPa 1200 3h-gl-6	25.47	0.75	9.81	4.54	2.44	5.65	2.58	0.32	47.96		0.02	0.10	99.64
LVP-5	MORB nopt 3.4 1600 2min-1	25.91	0.68	8.19	1.38	5.02	8.58	2.33	0.15	47.39		0.01	0.21	99.84
	MORB nopt 3.4 1600 2min-2	25.67	0.66	8.21	1.92	4.84	8.47	2.22	0.21	47.49		0.01	0.01	99.70
	MORB nopt 3.4 1600 2min-3	25.43	0.63	8.20	2.11	4.78	8.64	2.29	0.22	47.92		0.01	0.01	100.22
	MORB nopt 3.4 1600 2min-4	25.42	0.59	8.20	3.07	4.95	8.44	2.28	0.23	46.84		0.01	0.00	100.02
	MORB nopt 3.4 1600 2min-5	25.13	0.61	8.15	3.36	4.84	7.94	2.26	0.30	47.04		0.00	0.02	99.65
	MORB nopt 3.4 1600 2min-6	25.14	0.62	8.19	3.40	4.80	8.08	2.13	0.23	46.81		0.02	0.00	99.43
	MORB nopt 3.4 1600 2min-7	25.27	0.64	8.32	3.49	4.96	8.29	2.27	0.18	46.47		0.00	0.00	99.88
	MORB nopt 3.4 1600 2min-8	25.07	0.58	8.00	3.76	4.85	8.28	2.20	0.22	46.23		0.00	0.03	99.21
	MORB nopt 3.4 1600 2min-9	25.05	0.63	8.09	3.84	4.83	8.06	2.10	0.21	46.69		0.00	0.00	99.51
	MORB nopt 3.4 1600 2min-10	24.97	0.70	8.22	3.85	4.83	8.22	2.28	0.21	46.25		0.01	0.03	99.58
	MORB nopt 3.4 1600 2min-12	25.38	0.73	8.10	2.47	4.97	8.56	2.26	0.27	47.55		0.00	0.00	100.28
	MORB nopt 3.4 1600 2min-6	25.21	0.64	8.24	3.18	4.89	8.30	2.26	0.18	47.40		0.00	0.00	100.30
LVP-6	MORB in PtO2 3.4 1500 2min big Line 014	22.77	0.58	7.86	7.17	4.83	8.27	1.99	0.18	45.91		0.00	0.00	99.56
	MORB in PtO2 3.4 1500 2min big Line 011	22.68	0.56	7.92	7.80	4.63	7.80	1.91	0.21	46.24		0.00	0.00	99.74
	MORB in PtO2 3.4 1500 2min big Line 008	23.05	0.58	7.63	7.97	4.39	7.71	2.01	0.17	46.25		0.00	0.00	99.76
	MORB+O 3.4 1500 2min-5	22.91	0.61	7.63	8.11	4.35	7.93	1.86	0.17	46.22		0.00	0.00	99.79
	MORB in PtO2 3.4 1500 2min big Line 019	22.92	0.56	7.62	8.37	4.50	7.74	1.84	0.19	46.28		0.00	0.00	100.00
	MORB in PtO2 3.4 1500 2min big Line 018	22.76	0.59	7.58	8.19	4.57	7.94	2.05	0.16	46.29		0.00	0.00	100.13

Continued

Sample	Filename	Si	Ti	Al	Fe_T	Mg	Ca	Na	K	O	Re	Cl	Pt	Total
LVP-7	MORB+PtO2 3.4GPa-2 1600 2min-4	22.64	0.56	7.56	4.57	5.75	8.19	2.36	0.21	47.14		0.83	0.00	99.81
	MORB+PtO2 3.4GPa-2 1600 2min-6	22.59	0.54	7.71	4.54	5.65	8.06	2.13	0.21	47.11		0.80	0.07	99.40
	MORB+PtO2 3.4GPa-2 1600 2min-9	22.65	0.56	7.62	4.60	5.73	8.29	2.21	0.24	47.28		0.83	0.00	100.01
	MORB+PtO2 3.4GPa-2 1600 2min-14	22.60	0.53	7.71	4.78	5.89	8.10	2.30	0.20	47.54		0.81	0.07	100.53
	MORB+PtO2 3.4GPa-2 1600 2min-15	22.40	0.57	7.65	4.69	5.92	8.22	2.19	0.19	47.85		0.78	0.03	100.49
	MORB+PtO2 3.4GPa-2 1600 2min-16	22.49	0.54	7.60	4.84	6.03	8.24	2.21	0.17	46.57		0.85	0.17	99.71
	MORB+PtO2 3.4GPa-2 1600 2min-17	22.53	0.60	7.55	4.76	5.89	8.15	2.07	0.19	47.82		0.82	0.09	100.47
	MORB+PtO2 3.4GPa-2 1600 2min-18	22.79	0.58	7.62	4.79	6.03	8.20	2.26	0.23	47.01		0.79	0.16	100.46
	MORB+PtO2 3.4GPa-2 1600 2min-19	22.32	0.57	7.57	4.59	6.05	7.98	2.09	0.20	47.34		0.83	0.20	99.73
LVP-8	MORB+1%PtO2 3.4GPa 1700 4min-glass1	25.53	0.65	8.13	0.49	5.02	8.91	2.08	0.26	49.25		0.05	0.00	100.37
	MORB+1%PtO2 3.4GPa 1700 4min-glass2	25.29	0.65	8.28	0.56	5.04	9.03	2.09	0.26	48.51		0.05	0.01	99.78
	MORB+1%PtO2 3.4GPa 1700 4min-glass3	25.06	0.63	8.22	0.54	4.97	8.66	2.15	0.26	49.42		0.05	0.05	100.00
	MORB+1%PtO2 3.4GPa 1700 4min-glass4	25.03	0.71	7.93	0.53	5.02	9.08	2.19	0.19	48.67		0.04	0.00	99.38
LVP-10 (Cpx+Gl)	MORB nopt 5GPa 1450 1h-cpx13	23.59	0.25	7.60	7.11	5.67	9.23	2.09	0.01	43.97		0.00	0.00	99.50
	MORB nopt 5GPa 1450 1h-cpx14	23.97	0.35	7.86	6.94	5.45	8.91	2.29	0.03	44.58		0.00	0.00	100.38
	MORB nopt 5GPa 1450 1h-cpx15	23.87	0.38	7.74	6.59	5.43	8.82	2.12	0.04	45.45		0.00	0.00	100.44
	MORB nopt 5GPa 1450 1h-cpx11	23.60	0.32	7.87	6.55	5.91	9.46	2.05	0.04	45.36		0.01	0.00	101.16
	MORB nopt 5 1450 1h-gl9	27.13	0.88	8.92	0.65	3.86	7.88	2.22	0.34	48.71		0.03	0.00	100.61
	MORB nopt 5 1450 1h-gl7	27.34	0.83	8.87	0.69	3.98	7.73	1.96	0.38	48.55		0.02	0.00	100.36
	MORB nopt 5 1450 1h-gl11	27.40	0.93	8.78	0.76	3.68	7.54	2.06	0.34	48.79		0.02	0.00	100.30
	MORB nopt 5 1450 1h-gl4	27.06	0.88	8.78	0.58	3.94	7.70	2.05	0.36	48.56		0.02	0.03	99.95
LVP-11	MORB nopt 5 1600 2min-gl4	25.41	0.57	8.07	2.93	4.88	8.12	2.33	0.23	47.69		0.07	0.00	100.30
	MORB nopt 5 1600 2min-gl15	25.57	0.63	8.42	2.53	4.92	8.28	2.17	0.23	47.78		0.04	0.00	100.58
	MORB nopt 5 1600 2min-gl1	25.77	0.61	8.41	1.59	5.01	8.51	2.43	0.29	47.23		0.08	0.00	99.94
	MORB nopt 5 1600 2min-gl8	25.32	0.65	8.14	3.15	4.80	8.32	2.13	0.18	46.89		0.06	0.01	99.64
LVP-12 (Cpx+Coe+Gl)	MORB+satPtO2 5GPa 1400 1.5h-cpx1.1	23.47	0.16	8.10	6.84	5.30	9.23	2.43	0.00	44.04				99.57
	MORB+satPtO2 5GPa 1400 1.5h-cpx1.2	23.41	0.17	7.55	7.66	5.41	9.12	2.32	0.00	43.70				99.34
	MORB+satPtO2 5GPa 1400 1.5h-cpx1.3	23.65	0.13	7.54	6.77	5.58	9.07	2.57	0.02	43.87				99.21
	MORB+satPtO2 5GPa 1400 1.5h-cpx1.4	23.30	0.17	7.58	7.20	5.49	9.09	2.62	0.03	43.62				99.09
	MORB+satPtO2 5GPa 1400 1.5h-SiO2-1	46.80	0.04	0.08	0.00	0.01	0.01	0.00	0.00	53.42				100.35
	MORB+satPtO2 5GPa 1400 1.5h-SiO2-2	47.00	0.00	0.10	0.07	0.00	0.02	0.01	0.01	53.68				100.89
	MORB+satPtO2 5GPa 1400 1.5h-SiO2-3	46.99	0.02	0.07	0.00	0.00	0.03	0.00	0.00	53.63				100.75
	MORB+satPtO2 5GPa 1400 1.5h-SiO2-4	46.77	0.02	0.07	0.01	0.00	0.00	0.03	0.00	53.37				100.27
	MORB+satPtO2 5GPa 1400 1.5h-mid glass2	23.35	0.92	9.16	0.11	5.09	9.03	1.82	0.31	49.19		1.76	0.11	100.84
	MORB+satPtO2 5GPa 1400 1.5h-mid glass3	23.62	0.94	9.11	0.12	4.89	8.41	1.72	0.30	48.49		1.87	0.00	99.46
	MORB+satPtO2 5GPa 1400 1.5h-mid glass4	23.40	0.90	9.28	0.07	5.12	8.89	1.70	0.38	48.85		1.71	0.00	100.30
	MORB+satPtO2 5GPa 1400 1.5h-mid glass5	23.54	0.96	9.19	0.11	5.13	8.75	1.72	0.39	48.57		1.88	0.09	100.30

Continued

Sample	Filename	Si	Ti	Al	Fe_T	Mg	Ca	Na	K	O	Re	Cl	Pt	Total
LVP-13 (Cpx+Coe+Gl)	MORB+PtO2 5GPa 1450 1h bright cpx2	24.77	0.11	8.31	6.02	5.25	7.52	3.14	0.01	44.98				100.10
	MORB+PtO2 5GPa 1450 1h bright cpx3	24.60	0.10	8.05	6.25	5.19	8.18	3.18	0.01	45.06				100.64
	MORB+PtO2 5GPa 1450 1h bright cpx5	25.06	0.13	7.95	6.31	5.14	7.85	3.49	0.01	45.40				101.42
	MORB+PtO2 5GPa 1450 1h 5nA-Qz3	46.72	0.00	0.08	0.03	0.00	0.02	0.01	0.01	54.48				99.90
	MORB+PtO2 5GPa 1450 1h 5nA-Qz1	46.32	0.05	0.10	0.06	0.00	0.04	0.00	0.00	53.21				98.72
	MORB+PtO2 5GPa 1450 1h 5nA-Qz2	46.43	0.02	0.42	0.02	0.01	0.04	0.00	0.00	51.72				
	MORB+PtO2 5GPa 1450 1h 20um 3nA gl-1	23.68	1.03	7.59	1.37	4.14	9.06	1.88	0.41	49.27		1.58	0.00	100.02
	MORB+PtO2 5GPa 1450 1h 17um 3nA gl-2	23.36	0.90	7.75	1.38	4.24	9.06	1.87	0.42	49.51		1.53	0.20	100.21
	MORB+PtO2 5GPa 1450 1h 15um 5nA gl-1	23.51	0.91	7.86	1.47	4.15	8.79	1.74	0.35	49.16		1.60	0.00	99.54
	MORB+PtO2 5GPa 1450 1h 15um 5nA gl-2	23.40	0.96	7.84	1.40	4.16	9.00	1.81	0.42	49.13		1.56	0.00	99.68
	MORB+PtO2 5GPa 1450 1h 15um 5nA gl-3	23.56	0.90	7.97	1.39	4.17	8.77	1.81	0.41	49.49		1.50	0.14	100.11
	MORB+PtO2 5GPa 1450 1h 15um 5nA gl-4	23.46	0.92	7.74	1.54	4.16	9.05	1.84	0.45	48.77		1.65	0.01	99.57
LVP-14	MORB+PtO2 5GPa 1600 2min-1.1	25.20	0.53	7.79	2.70	4.37	7.93	2.25	0.25	48.67		0.18	0.00	99.86
	MORB+PtO2 5GPa 1600 2min-1.2	25.45	0.52	7.99	3.34	4.53	7.99	2.26	0.25	48.11		0.15	0.00	100.59
	MORB+PtO2 5GPa 1600 2min-1.3	24.98	0.57	7.88	3.57	4.46	7.79	2.10	0.25	47.72		0.23	0.08	99.61
	MORB+PtO2 5GPa 1600 2min-1.4	24.33	0.53	7.86	3.64	4.66	8.07	2.04	0.17	48.40		0.26	0.00	99.96
	MORB+PtO2 5GPa 1600 2min-1.5	23.84	0.59	8.27	3.80	4.81	8.06	1.93	0.22	48.40		0.28	0.04	100.23
LVP-15	MORB+PtO2 5GPa 1700-4.3	24.46	0.65	8.10	2.62	4.76	7.98	2.08	0.23	48.99		0.37	0.00	100.24
	MORB+PtO2 5GPa 1700-4.4	24.78	0.56	8.00	2.64	4.88	8.25	2.21	0.25	48.17		0.35	0.00	100.07
	MORB+PtO2 5GPa 1700-1.6	24.11	0.60	8.09	2.62	4.75	8.17	1.97	0.22	48.87		0.35	0.03	99.77
	MORB+PtO2 5GPa 1700-2.2	24.55	0.55	8.27	2.64	4.79	8.25	2.17	0.23	47.87		0.38	0.00	99.69
	MORB+PtO2 5GPa 1700-4.1	24.28	0.56	8.02	2.59	4.86	8.35	2.31	0.30	47.90		0.35	0.01	99.52
LVP-16	MORB nopt 7 1600 5min-2	25.30	0.66	8.20	1.95	4.98	8.56	2.20	0.30	47.20		0.00	0.00	99.66
	MORB nopt 7 1600 5min-3	25.61	0.62	8.12	1.83	4.86	8.62	2.10	0.19	47.30		0.01	0.00	99.34
	MORB nopt 7 1600 5min-6	25.49	0.66	8.20	1.93	4.92	8.52	2.37	0.23	47.55		0.01	0.04	99.26
	MORB nopt 7 1600 5min-7	25.46	0.66	8.17	1.99	4.80	8.49	2.15	0.22	47.40		0.01	0.00	99.12
	MORB nopt 7 1600 5min-9	25.52	0.68	8.20	1.78	5.05	8.52	2.18	0.25	47.34		0.00	0.00	100.38
LVP-17 (Cpx+Gt+Coe+Gl)	MORB+sat PtO2 7GPa 1400 1.5h-cpx core1	25.39	0.13	7.98	5.70	5.53	6.95	3.36	0.02	45.41		0.00		100.45
	MORB+sat PtO2 7GPa 1400 1.5h-cpx core2	25.08	0.07	7.97	5.41	5.34	7.14	3.51	0.01	46.34		0.00		101.00
	MORB+sat PtO2 7GPa 1400 1.5h-cpx core3	25.26	0.12	7.97	5.72	5.58	6.78	3.21	0.00	46.13		0.00		100.77
	MORB+sat PtO2 7GPa 1400 1.5h-cpx core4	25.08	0.07	8.10	5.35	5.57	6.37	3.16	0.00	44.43		0.01		98.13
	MORB+sat PtO2 7GPa 1400 1.5h-Fe Grt1	19.75	0.20	12.14	10.32	9.03	5.59	0.13	0.00	42.61		0.00		99.77
	MORB+sat PtO2 7GPa 1400 1.5h-Fe Grt2	19.53	0.22	11.85	10.30	9.35	5.22	0.08	0.00	43.69		0.00		100.37
	MORB+sat PtO2 7GPa 1400 1.5h-Fe Grt3	19.59	0.21	11.82	10.10	9.59	5.22	0.12	0.00	42.97		0.00		99.70
	MORB+sat PtO2 7GPa 1400 1.5h-Fe Grt4	19.67	0.25	11.71	10.24	9.46	5.03	0.12	0.01	43.89		0.00		100.39
	MORB+sat PtO2 7GPa 1400 1.5h-Fe Grt5	19.44	0.19	11.85	10.13	9.56	5.14	0.16	0.00	43.62		0.01		100.09
	MORB+sat PtO2 7GPa 1400 1.5h-SiO2-3	45.95	0.04	0.11	0.02	0.01	0.08	0.00	0.00	54.10		0.02		100.42
	MORB+sat PtO2 7GPa 1400 1.5h-SiO2-9	45.94	0.00	0.14	0.06	0.02	0.10	0.02	0.01	54.05		0.03		100.37
	MORB+sat PtO2 7GPa 1400 1.5h-SiO2-2	46.32	0.01	0.09	0.03	0.00	0.05	0.01	0.02	53.26		0.00		99.94
	MORB+sat PtO2 7GPa 1400 1.5h-SiO2-5	46.36	0.00	0.09	0.02	0.00	0.07	0.03	0.04	52.95		0.00		99.76
	MORB+sat PtO2 7GPa 1400 1.5h-SiO2-7	45.55	0.03	0.10	0.03	0.00	0.04	0.01	0.00	53.82		0.00		99.68
	MORB+sat PtO2 7GPa 1400 1.5h-gl7	21.87	1.53	7.22	0.97	3.80	9.69	1.81	0.66	51.20		1.64	0.10	100.49
	MORB+sat PtO2 7GPa 1400 1.5h-gl9	21.73	1.47	7.09	1.00	3.84	9.45	1.69	0.60	51.68		1.64	0.01	100.20
	MORB+sat PtO2 7GPa 1400 1.5h-gl10	22.47	1.43	7.24	1.05	3.70	9.38	1.63	0.55	50.55		1.61	0.00	99.61
	MORB+sat PtO2 7GPa 1400 1.5h-gl8	22.01	1.52	7.16	0.97	3.93	9.75	1.75	0.60	50.28		1.59	0.00	99.56

Continued

Sample	Filename	Si	Ti	Al	Fe_T	Mg	Ca	Na	K	O	Re	Cl	Pt	Total
LVP-18 (Cpx+Gt+Coe+Gl)	MORB+sat PtO2 7GPa 1450 1h-cpx core1.1	25.31	0.16	8.60	5.50	5.03	7.35	3.16	0.01	45.28		0.00	0.00	100.41
	MORB+sat PtO2 7GPa 1450 1h-cpx core1.2	25.15	0.19	9.04	4.00	5.07	7.26	3.12	0.01	45.53		0.01	0.00	99.39
	MORB+sat PtO2 7GPa 1450 1h-cpx core1.3	25.22	0.15	8.79	4.52	4.37	7.42	3.36	0.03	45.81		0.00	0.00	99.69
	MORB+sat PtO2 7GPa 1450 1h-cpx core2.1	25.16	0.15	8.38	5.47	4.97	7.40	3.07	0.03	45.50		0.00	0.00	100.12
	MORB+sat PtO2 7GPa 1450 1h-cpx core2.2	25.43	0.14	8.84	4.66	4.72	6.79	3.35	0.03	46.22		0.01	0.00	100.18
	MORB+sat PtO2 7GPa 1450 1h-grt core1	19.41	0.27	12.08	9.27	9.46	5.01	0.11	0.00	44.34		0.00	0.03	99.97
	MORB+sat PtO2 7GPa 1450 1h-grt core2	19.42	0.25	12.09	9.63	9.52	4.73	0.13	0.00	44.11		0.00	0.01	99.90
	MORB+sat PtO2 7GPa 1450 1h-grt core3	18.94	0.26	11.80	10.88	8.91	5.21	0.11	0.00	43.05		0.00	0.05	99.20
	MORB+sat PtO2 7GPa 1450 1h-grt core4	19.26	0.31	11.57	9.83	9.76	5.26	0.12	0.00	43.60		0.00	0.03	99.74
	MORB+sat PtO2 7GPa 1450 1h-grt core5	18.77	0.29	11.71	10.60	8.59	5.17	0.14	0.01	44.10		0.02	0.00	99.39
	MORB+sat PtO2 7GPa 1450 1h-grt core6	19.38	0.42	11.42	9.81	9.25	5.38	0.10	0.00	44.59		0.01	0.12	100.49
	MORB+sat PtO2 7GPa 1450 1h-grt core7	19.28	0.36	11.54	10.83	8.89	5.47	0.11	0.03	43.75		0.00	0.01	100.29
	MORB+sat PtO2 7GPa 1450 1h-SiO2-1	46.63	0.02	0.06	0.08	0.00	0.05	0.02	0.03	54.36		0.01	0.05	101.31
	MORB+sat PtO2 7GPa 1450 1h-SiO2-3	46.60	0.00	0.07	0.04	0.00	0.03	0.07	0.01	54.42		0.01	0.07	101.32
	MORB+sat PtO2 7GPa 1450 1h-SiO2-4	46.12	0.01	0.08	0.11	0.00	0.01	0.01	0.00	52.97		0.01	0.00	99.32
	MORB+sat PtO2 7GPa 1450 1h-SiO2-5	46.46	0.02	0.09	0.06	0.00	0.02	0.00	0.00	52.74		0.01	0.09	99.50
	MORB+sat PtO2 7GPa 1450 1h-gl-1	21.56	1.64	7.11	1.16	4.10	10.12	1.68	0.60	50.28		2.27	0.00	100.52
	MORB+sat PtO2 7GPa 1450 1h-gl-2	21.42	1.55	7.12	1.13	4.16	10.19	1.69	0.54	50.04		2.30	0.00	100.14
	MORB+sat PtO2 7GPa 1450 1h-gl-3	21.49	1.54	7.09	1.10	4.12	10.26	1.62	0.67	50.37		2.25	0.00	100.52
	MORB+sat PtO2 7GPa 1450 1h-gl-4	21.72	1.63	7.14	1.13	4.20	10.42	1.64	0.52	50.27		2.33	0.00	100.99
	MORB+sat PtO2 7GPa 1450 1h-gl-5	21.45	1.61	7.07	1.13	4.34	10.30	1.61	0.63	50.19		2.24	0.06	100.63
	MORB+sat PtO2 7GPa 1450 1h-gl-6	21.85	1.67	7.13	1.14	4.19	10.20	1.83	0.46	49.68		2.34	0.11	100.59
	MORB+sat PtO2 7GPa 1450 1h-gl-9	21.92	1.53	7.29	1.13	4.40	10.18	1.77	0.60	49.06		2.25	0.00	100.12
	MORB+sat PtO2 7GPa 1450 1h-gl-10	21.69	1.66	7.26	1.06	4.32	10.36	1.57	0.56	49.75		2.19	0.02	100.45
LVP-19	MORB+PtO2 7GPa 1600 2min-1	25.32	0.52	7.72	3.53	3.81	7.62	2.02	0.16	48.82		0.13	0.05	99.70
	MORB+PtO2 7GPa 1600 2min-2	23.31	0.60	7.60	4.95	4.53	7.79	1.92	0.16	48.30		0.17	0.00	99.33
	MORB+PtO2 7GPa 1600 2min-3	23.11	0.57	7.72	5.32	4.38	7.69	1.97	0.14	47.45		0.18	0.00	98.51
	MORB+PtO2 7GPa 1600 2min-4	22.92	0.67	8.29	4.93	4.75	8.02	1.82	0.17	47.84		0.20	0.06	99.68
	MORB+PtO2 7GPa 1600 2min-5	23.01	0.61	7.94	7.32	4.57	7.67	1.84	0.24	46.80		0.28	0.19	100.46
LVP-20	MORB+PtO2 7GPa 1700 2min-2	24.74	0.64	8.31	2.55	4.72	8.12	1.94	0.22	49.22		0.19	0.06	100.72
	MORB+PtO2 7GPa 1700 2min-3	25.04	0.61	8.23	2.86	4.74	8.24	1.90	0.19	48.58		0.15	0.00	100.53
	MORB+PtO2 7GPa 1700 2min-4	25.20	0.63	8.23	2.82	4.66	7.99	1.90	0.21	48.39		0.13	0.09	100.24
	MORB+PtO2 7GPa 1700 2min-2	24.74	0.64	8.31	2.55	4.72	8.12	1.93	0.22	48.70		0.19	0.06	100.18
	MORB+PtO2 7GPa 1700 2min-3	25.04	0.61	8.22	2.86	4.73	8.24	1.90	0.19	47.74		0.15	0.00	99.68
LVP-21	C20 MORB+3%PtO2 7GPa 1700 4min-glass8	24.43	0.63	7.88	1.57	4.66	8.41	2.12	0.24	49.42		0.22	0.00	99.58
	C20 MORB+3%PtO2 7GPa 1700 4min-glass9	24.53	0.66	7.90	1.59	4.65	8.31	2.12	0.28	49.47		0.18	0.01	99.69
	C20 MORB+3%PtO2 7GPa 1700 4min-glass11	24.40	0.62	7.96	1.50	4.66	8.45	2.20	0.28	49.71		0.22	0.10	100.10
	C20 MORB+3%PtO2 7GPa 1700 4min-glass9	24.53	0.66	7.91	1.59	4.65	8.31	2.13	0.28	50.03		0.18	0.01	100.26
	C20 MORB+3%PtO2 7GPa 1700 4min-glass6	24.58	0.69	7.92	1.23	4.72	8.62	2.24	0.24	49.97		0.20	0.00	100.40
LVP-22	MORB+PtO2 7GPa 1800 4min-1	23.88	0.56	7.56	4.02	4.18	7.39	2.06	0.20	50.36		0.18	0.06	100.44
	MORB+PtO2 7GPa 1800 4min-3	24.09	0.59	7.56	4.02	4.07	7.30	1.81	0.18	50.08		0.19	0.06	99.94
	MORB+PtO2 7GPa 1800 4min-4	24.23	0.55	7.48	3.96	4.30	7.31	1.79	0.19	49.44		0.18	0.05	99.48

Table S3. Summary of individual SIMS analysis at IGGCAS for experimental run products of this study.

Sample	Filename	^{1}H Counts	^{1}H Rate	^{12}C Counts	^{12}C Rate	^{18}O Counts	^{18}O Rate	$^{1}H/^{18}O$ Ratio	$H/^{18}O$ Err Mea	$H/^{18}O$ Poisson	$^{12}C/^{18}O$ Ratio	$C/^{18}O$ Err Mea	$^{12}C/^{18}O$ Poisson		H_2O (ppm)		CO_2 (ppm)
MORB starting comp.	20231010-MORB-origin_	8.83E+05	1.15E+04	1.59E+04	2.08E+02	1.37934E+007	1.80E+05	6.40E-02	8.210E-001	1.10E-01	1.16E-03	9.86E-01	7.92E-01		1265		25
	20231008-MORB-origin_	4.75E+05	6.19E+03	2.62E+04	3.41E+02	2.17051E+007	2.83E+05	2.19E-02	3.780E-001	1.47E-01	1.21E-03	8.16E-01	6.18E-01		433		26
	20231008-MORB-origin_	4.53E+05	5.90E+03	2.33E+04	3.04E+02	2.45323E+007	3.19E+05	1.85E-02	2.720E-001	1.50E-01	9.50E-04	8.06E-01	6.55E-01		365		20
	20231008-MORB-origin_	4.01E+05	5.22E+03	2.31E+04	3.00E+02	2.32083E+007	3.02E+05	1.73E-02	2.360E-001	1.59E-01	9.94E-04	8.34E-01	6.59E-01		341		21
	20231008-MORB-origin_	2.77E+05	3.61E+03	1.71E+04	2.22E+02	1.54147E+007	2.01E+05	1.80E-02	2.960E-001	1.92E-01	1.11E-03	8.59E-01	7.66E-01		355		24
	20231010-MORB-origin_	2.10E+05	2.73E+03	1.92E+04	2.50E+02	1.02056E+007	1.33E+05	2.06E-02	2.490E-001	2.21E-01	1.88E-03	8.33E-01	7.22E-01		406		41
														Ave.	**380**	Ave.	**26**
														SD	**38**	SD	**7**
LVP-6	20231009-MO3-41500_1	1.39E+07	1.81E+05	1.41E+07	1.84E+05	4.04269E+007	5.26E+05	3.44E-01	4.730E-002	3.11E-02	3.50E-01	3.62E-02	3.09E-02		6794		7538
	20231009-MO3-41500_4	1.43E+07	1.86E+05	1.50E+07	1.96E+05	4.09014E+007	5.33E+05	3.48E-01	4.860E-002	3.08E-02	3.67E-01	4.01E-02	3.02E-02		6887		7923
														Ave.	**6840**	Ave.	**7730**
														SD	**65**	SD	**272**
LVP-7	20231009-MO3-41600_2	1.88E+07	2.45E+05	1.32E+07	1.71E+05	3.05556E+007	3.98E+05	6.16E-01	3.650E-002	2.93E-02	4.31E-01	8.08E-02	3.30E-02		12170		9288
	20231009-MO3-41600_3	2.10E+07	2.73E+05	1.38E+07	1.80E+05	3.24901E+007	4.23E+05	6.45E-01	3.090E-002	2.80E-02	4.25E-01	7.31E-02	3.21E-02		12753		9157
														Ave.	**12462**	Ave.	**9223**
														SD	**413**	SD	**92**
LVP-14	20231009-MO51600RC_	1.41E+07	1.83E+05	1.95E+07	2.53E+05	3.16062E+007	4.12E+05	4.46E-01	6.220E-002	3.20E-02	6.16E-01	7.77E-02	2.88E-02		8808		13273
	20231009-MO51600RC_	1.39E+07	1.81E+05	2.00E+07	2.60E+05	3.25805E+007	4.24E+05	4.27E-01	4.930E-002	3.20E-02	6.14E-01	8.15E-02	2.84E-02		8443		13237
	20231009-MO51600RC_	1.54E+07	2.00E+05	1.91E+07	2.49E+05	3.24007E+007	4.22E+05	4.74E-01	4.690E-002	3.10E-02	5.91E-01	7.34E-02	2.88E-02		9366		12741
	20231009-MO51600RC_	1.27E+07	1.66E+05	1.91E+07	2.48E+05	3.06936E+007	4.00E+05	4.15E-01	6.790E-002	3.33E-02	6.21E-01	1.10E-01	2.92E-02		8197		13384
	20231009-MO51600RC_	1.24E+07	1.61E+05	1.74E+07	2.26E+05	3.01408E+007	3.92E+05	4.11E-01	3.610E-002	3.37E-02	5.77E-01	7.90E-02	3.01E-02		8126		12439
	20231009-MO51600RC_	1.18E+07	1.54E+05	1.83E+07	2.39E+05	2.89527E+007	3.77E+05	4.09E-01	6.950E-002	3.45E-02	6.33E-01	9.12E-02	2.98E-02		8077		13654
	20231009-MO51600RC_	1.33E+07	1.73E+05	1.91E+07	2.49E+05	3.25234E+007	4.23E+05	4.08E-01	4.120E-002	3.26E-02	5.88E-01	7.62E-02	2.88E-02		8053		12686
														Ave.	**8439**	Ave.	**13059**
														SD	**489**	SD	**440**
LVP-15	20231009-MO51700_3	1.01E+07	1.31E+05	1.07E+07	1.40E+05	2.23114E+007	2.91E+05	4.51E-01	9.770E-002	3.80E-02	4.82E-01	1.72E-01	3.71E-02		8909		10386
	20231009-MO51700_4	8.55E+06	1.11E+05	8.35E+06	1.09E+05	1.84076E+007	2.40E+05	4.64E-01	9.930E-002	4.14E-02	4.53E-01	1.50E-01	4.17E-02		9177		9776
														Ave.	**9043**	Ave.	**10081**
														SD	**190**	SD	**431**
LVP-22	20231009-MO71800_1	5.97E+06	7.77E+04	1.86E+07	2.42E+05	2.06256E+007	2.69E+05	2.89E-01	4.040E-001	4.65E-02	9.00E-01	6.88E-01	3.20E-02		5718		19401
	20231009-MO71800_2	7.33E+06	9.55E+04	1.83E+07	2.39E+05	3.09240E+007	4.03E+05	2.37E-01	1.100E-001	4.11E-02	5.93E-01	1.15E-01	2.95E-02		4685		12794
	20231009-MO71800_3	1.14E+07	1.49E+05	2.47E+07	3.21E+05	3.29481E+007	4.29E+05	3.47E-01	8.790E-002	3.43E-02	7.49E-01	2.44E-01	2.66E-02		6849		16159
	20231009-MO71800_6	8.60E+06	1.12E+05	2.28E+07	2.97E+05	3.21635E+007	4.19E+05	2.67E-01	9.210E-002	3.84E-02	7.09E-01	7.43E-02	2.74E-02		5286		15299
														Ave.	**5635**	Ave.	**15913**
														SD	**914**	SD	**2729**
LVP-2	20231219-C1_1 0.5GPa	3.69E+06	1.71E+04	1.27E+05	5.89E+02	1.14029E+007	5.27E+04	3.24E-01	6.460E-002	5.99E-02	1.12E-02	3.08E-01	2.82E-01		5553		258
	20231219-C1_2 0.5GPa	9.50E+06	4.39E+04	3.00E+05	1.39E+03	2.31924E+007	1.07E+05	4.10E-01	4.760E-002	3.85E-02	1.29E-02	2.70E-01	1.84E-01		7021		298
															**6287**		**278**
															**1038**		**29**
LVP-3	20231219-C2_1 0.5GPa	6.99E+07	3.23E+05	1.55E+06	7.15E+03	6.31179E+007	2.92E+05	1.11E+00	4.240E-002	1.74E-02	2.45E-02	8.94E-02	8.14E-02		18973		565
	20231219-C2_2 0.5GPa	7.17E+07	3.31E+05	1.47E+06	6.81E+03	6.42784E+007	2.97E+05	1.12E+00	3.020E-002	1.72E-02	2.29E-02	1.02E-01	8.33E-02		19118		529
														Ave.	**19045**	Ave.	**547**
														SD	**102**	SD	**26**
LVP-8	20231219-C7_1 3.4GPa	1.56E+07	7.22E+04	1.99E+07	9.22E+04	5.53706E+007	2.56E+05	2.82E-01	1.390E-001	2.87E-02	3.60E-01	2.05E-01	2.61E-02		4833		8310
	20231219-C7_2 3.4GPa	2.04E+07	9.43E+04	2.37E+07	1.10E+05	6.70955E+007	3.10E+05	3.04E-01	1.050E-001	2.53E-02	3.53E-01	1.56E-01	2.39E-02		5211		8153
	20231219-C7_3 3.4GPa	1.99E+07	9.18E+04	2.34E+07	1.08E+05	6.59367E+007	3.05E+05	3.01E-01	1.060E-001	2.56E-02	3.55E-01	1.89E-01	2.41E-02		5161		8194
														Ave.	**5068**	Ave.	**8219**
														SD	**205**	SD	**82**
LVP-13	20231219-C12gl_1	1.62E+07	7.49E+04	8.63E+06	3.99E+04	1.77321E+007	8.19E+04	9.15E-01	6.020E-002	3.44E-02	4.87E-01	6.67E-02	4.15E-02		15675		11227
	20231219-C12gl_2	2.55E+07	1.18E+05	1.43E+07	6.62E+04	2.57496E+007	1.19E+05	9.90E-01	4.210E-002	2.79E-02	5.56E-01	6.60E-02	3.30E-02		16972		12829
	20231219-C12gl_3	4.03E+07	1.86E+05	2.78E+07	1.29E+05	4.47217E+007	2.07E+05	9.01E-01	2.620E-002	2.17E-02	6.22E-01	8.61E-02	2.41E-02		15437		14354
	20231219-C12gl_4	3.93E+07	1.82E+05	2.78E+07	1.29E+05	4.43237E+007	2.05E+05	8.86E-01	2.460E-002	2.19E-02	6.28E-01	7.39E-02	2.42E-02		15191		14494
														Ave.	**15819**	Ave.	**13226**
														SD	**794**	SD	**1531**
LVP-19	20231219-C17_1 7GPa	3.08E+07	1.42E+05	5.21E+07	2.41E+05	7.48866E+007	3.46E+05	4.11E-01	3.140E-002	2.14E-02	6.96E-01	3.43E-02	1.80E-02		7051		16057
	20231219-C17_3 7GPa	1.86E+07	8.60E+04	2.85E+07	1.32E+05	4.16581E+007	1.93E+05	4.47E-01	5.090E-002	2.79E-02	6.85E-01	8.48E-02	2.43E-02		7654		15791
														Ave.	**7352**	Ave.	**15924**
														SD	**426**	SD	**188**
LVP-20	20231219-C18_1 7GPa	1.68E+07	7.76E+04	2.80E+07	1.29E+05	4.90363E+007	2.27E+05	3.42E-01	5.940E-002	2.83E-02	5.70E-01	7.10E-02	2.37E-02		5867		13154
	20231219-C18_2 7GPa 1	2.54E+07	1.17E+05	4.01E+07	1.85E+05	6.57407E+007	3.04E+05	3.86E-01	6.620E-002	2.34E-02	6.09E-01	1.04E-01	2.00E-02		6610		14058
	20231219-C18_3 7GPa 1	2.47E+07	1.14E+05	3.88E+07	1.79E+05	6.47246E+007	2.99E+05	3.82E-01	9.160E-002	2.36E-02	6.00E-01	7.24E-02	2.03E-02		6551		13841
														Ave.	**6343**	Ave.	**13684**
														SD	**413**	SD	**472**
LVP-21	20231219-C20_1 7GPa 3	10745200	49654.3	21509700	99397.9	3.95520E+007	182773	0.271671	5.470E-002	3.44E-02	5.44E-01	9.51E-02	2.68E-02		4656		12545
	20231219-C20_2 7GPa 3	10103600	46689.5	19100100	88262.9	3.71619E+007	171728	0.27188	5.000E-002	3.55E-02	5.14E-01	1.04E-01	2.82E-02		4659		11856
	20231219-C20_3 7GPa 3	10745200	49654.3	23893500	110414	4.26384E+007	197035	0.252008	5.320E-002	3.41E-02	5.60E-01	8.73E-02	2.56E-02		4319		12926
														Ave.	**4544**	Ave.	**12442**
														SD	**196**	SD	**543**

Ave, average
SD, standard deviation

Table S4. Summary of individual SIMS analysis at GIGCAS for experimental run products of this study.

Sample	Filename	^{12}C Counts	^{12}C Rate	^{16}OH Counts	^{16}OH Rate	^{30}Si Counts	^{30}Si Rate	^{12}C/^{30}Si Ratio	^{12}C/^{30}Si Err Mean	^{12}C/^{30}Si Poisson	^{16}OH/^{30}Si Ratio	^{16}OH/^{30}Si Err Mea	^{16}OH/^{30}Si Poisson	SiO_2 (wt.%)	H/Si*SiO_2(%)		**H_2O (ppm)**	C/Si*SiO_2(%)		**CO_2 (ppm)**
LVP-1		2.44E+02	8.41E+01	6.21E+03	2.14E+03	6.16E+05	2.12E+05	6.30E+01	2.17E+01	3.96E-04	8.25E+00	6.40E+00	1.01E-02	50.05	5.06E-03		95	1.99E-04		-21
		3.00E+02	1.03E+02	6.09E+03	2.10E+03	6.37E+05	2.20E+05	5.80E+01	2.00E+01	4.71E-04	5.37E+00	5.78E+00	9.57E-03	50.05	4.80E-03		90	2.36E-04		-19
		3.13E+02	1.08E+02	6.12E+03	2.11E+03	6.43E+05	2.22E+05	4.30E+01	1.48E+01	4.86E-04	6.06E+00	5.65E+00	9.51E-03	50.05	4.76E-03		89	2.44E-04		-19
		2.79E+02	9.62E+01	6.20E+03	2.14E+03	6.40E+05	2.21E+05	3.60E+01	1.24E+01	4.36E-04	5.10E+00	5.99E+00	9.70E-03	50.05	4.86E-03		91	2.18E-04		-20
																Ave.	**91**		Ave.	**-20**
																SD.	**3**		SD.	**1**
LVP-4	20240508_S3410_M32-nopt11200-gl_3	1.02E+05	3.52E+04	1.02E+06	3.52E+05	4.84E+05	1.67E+05	2.11E-01	3.39E-01	3.45E-01	2.11E+00	1.75E-01	1.75E-01	54.57	1.15E+00		23966	1.15E-01		4258
	20240508_S3410_M32-nopt11200-gl_4	1.16E+05	3.99E+04	1.13E+06	3.91E+05	5.02E+05	1.73E+05	2.31E-01	2.86E-01	3.26E-01	2.26E+00	1.53E-01	1.70E-01	54.57	1.23E+00		25664	1.26E-01		4660
																Ave.	**24815**		Ave.	**4459**
																SD.	**1200**		SD.	**285**
LVP-5	20240506_S3408_M35_noPt31600gl_1	3.86E+05	1.33E+05	2.58E+05	8.90E+04	5.89E+05	2.03E+05	6.56E-01	2.23E-01	2.07E-01	4.38E-01	3.47E-01	2.36E-01	54.13	2.19E-01		4796	3.55E-01		12997
	20240506_S3408_M35_noPt31600gl_2	4.30E+05	1.48E+05	2.53E+05	8.71E+04	5.93E+05	2.04E+05	7.26E-01	2.43E-01	2.00E-01	4.26E-01	4.49E-01	2.38E-01	54.13	2.13E-01		4662	3.93E-01		14398
	20240506_S3408_M35_noPt31600gl_3	4.53E+05	1.56E+05	2.50E+05	8.62E+04	5.84E+05	2.01E+05	7.76E-01	2.01E-01	1.98E-01	4.28E-01	4.51E-01	2.39E-01	54.13	2.14E-01		4688	4.20E-01		15375
	20240506_S3408_M35_noPt31600gl_4	4.56E+05	1.57E+05	2.37E+05	8.17E+04	5.50E+05	1.90E+05	8.28E-01	2.08E-01	2.00E-01	4.30E-01	4.95E-01	2.46E-01	54.13	2.15E-01		4709	4.48E-01		16419
	20240506_S3408_M35_noPt31600gl_5	4.16E+05	1.43E+05	2.01E+05	6.92E+04	5.23E+05	1.80E+05	7.94E-01	1.98E-01	2.08E-01	3.83E-01	5.97E-01	2.63E-01	54.13	1.92E-01		4193	4.30E-01		15744
																Ave.	**4609**		Ave.	**14986**
								7.94E-01								SD.	**238**		SD.	**1331**
LVP-12	20240430_S3408_M13sa51400gl_1	4.04E+05	1.39E+05	6.36E+05	2.19E+05	3.91E+05	1.35E+05	1.03E+00	2.34E-01	2.24E-01	1.63E+00	2.15E-01	2.03E-01	54.32	8.84E-01		15526	5.62E-01		23489
	20240430_S3408_M13sa51400gl_2	3.69E+05	1.27E+05	5.91E+05	2.04E+05	3.59E+05	1.24E+05	1.03E+00	2.57E-01	2.35E-01	1.65E+00	2.43E-01	2.12E-01	54.32	8.94E-01		15713	5.58E-01		23336
	20240430_S3408_M13sa51400gl_3	4.09E+05	1.41E+05	6.45E+05	2.22E+05	3.93E+05	1.36E+05	1.04E+00	2.18E-01	2.23E-01	1.64E+00	1.84E-01	2.02E-01	54.32	8.91E-01		15647	5.66E-01		23636
	20240430_S3408_M13sa51400gl_4	3.97E+05	1.37E+05	6.35E+05	2.19E+05	3.84E+05	1.32E+05	1.04E+00	2.42E-01	2.26E-01	1.66E+00	2.08E-01	2.04E-01	54.32	8.99E-01		15801	5.63E-01		23519
	20240430_S3408_M13sa51400gl_5	3.99E+05	1.38E+05	6.26E+05	2.16E+05	3.81E+05	1.31E+05	1.05E+00	2.53E-01	2.26E-01	1.64E+00	2.12E-01	2.05E-01	54.32	8.93E-01		15688	5.69E-01		23777
																Ave.	**15675**		Ave.	**23551**
																SD.	**101**		SD.	**165**
LVP-10	20240430_S3408_M18nopt51450gl_1	3.28E+05	1.13E+05	3.79E+05	1.31E+05	4.24E+05	1.46E+05	7.73E-01	2.40E-01	2.33E-01	8.94E-01	2.36E-01	2.24E-01	58.26	5.21E-01		9147	4.50E-01		18815
	20240430_S3408_M18nopt51450gl_2	3.54E+05	1.22E+05	3.95E+05	1.36E+05	4.45E+05	1.53E+05	7.94E-01	2.31E-01	2.25E-01	8.87E-01	1.72E-01	2.19E-01	58.26	5.17E-01		9076	4.63E-01		19337
	20240430_S3408_M18nopt51450gl_3	3.39E+05	1.17E+05	3.82E+05	1.32E+05	4.34E+05	1.50E+05	7.81E-01	2.18E-01	2.29E-01	8.81E-01	2.30E-01	2.22E-01	58.26	5.13E-01		9017	4.55E-01		18999
	20240430_S3408_M18nopt51450gl_4	3.47E+05	1.20E+05	3.85E+05	1.33E+05	4.28E+05	1.48E+05	8.11E-01	2.10E-01	2.28E-01	8.99E-01	1.63E-01	2.22E-01	58.26	5.24E-01		9200	4.73E-01		19746
																Ave.	**9110**		Ave.	**19224**
																SD.	**80**		SD.	**410**
LVP-11	20240430_S3408_M19nopt51600gl_1	3.32E+05	1.14E+05	3.52E+05	1.21E+05	4.47E+05	1.54E+05	7.44E-01	2.18E-01	2.29E-01	7.88E-01	2.26E-01	2.25E-01	54.48	4.29E-01		7540	4.05E-01		16921
	20240430_S3408_M19nopt51600gl_2	3.23E+05	1.11E+05	3.53E+05	1.22E+05	4.46E+05	1.54E+05	7.23E-01	2.64E-01	2.31E-01	7.91E-01	2.92E-01	2.25E-01	54.48	4.31E-01		7571	3.94E-01		16453
	20240430_S3408_M19nopt51600gl_3	3.21E+05	1.11E+05	3.55E+05	1.22E+05	4.48E+05	1.55E+05	7.17E-01	2.74E-01	2.31E-01	7.92E-01	2.61E-01	2.25E-01	54.48	4.32E-01		7580	3.91E-01		16325
	20240430_S3408_M19nopt51600gl_4	3.30E+05	1.14E+05	3.66E+05	1.26E+05	4.73E+05	1.63E+05	6.97E-01	1.92E-01	2.27E-01	7.74E-01	2.22E-01	2.20E-01	54.48	4.22E-01		7404	3.80E-01		15854
	20240430_S3408_M19nopt51600gl_5	3.42E+05	1.18E+05	3.64E+05	1.26E+05	4.71E+05	1.63E+05	7.25E-01	2.09E-01	2.25E-01	7.72E-01	2.47E-01	2.21E-01	54.48	4.21E-01		7388	3.95E-01		16501
	20240430_S3408_M19nopt51600gl_6	3.23E+05	1.11E+05	3.50E+05	1.21E+05	4.48E+05	1.54E+05	7.21E-01	2.48E-01	2.31E-01	7.82E-01	2.13E-01	2.26E-01	54.48	4.26E-01		7480	3.93E-01		16402
																Ave.	**7494**		Ave.	**16409**
																SD.	**84**		SD.	**343**
LVP-17	20240430_S3408_M22sa71400gl_1	7.35E+05	2.54E+05	1.66E+06	5.72E+05	3.44E+05	1.19E+05	2.14E+00	2.17E-01	2.07E-01	4.82E+00	1.90E-01	1.87E-01	47.11	2.27E+00		39913	1.01E+00		42087
	20240430_S3408_M22sa71400gl_2	5.75E+05	1.98E+05	1.20E+06	4.15E+05	2.63E+05	9.07E+04	2.19E+00	2.40E-01	2.35E-01	4.57E+00	2.09E-01	2.15E-01	47.11	2.15E+00		37846	1.03E+00		43097
	20240430_S3408_M22sa71400gl_3	7.41E+05	2.56E+05	1.71E+06	5.91E+05	3.50E+05	1.21E+05	2.12E+00	1.85E-01	2.05E-01	4.90E+00	1.82E-01	1.86E-01	47.11	2.31E+00		40552	9.98E-01		41741
	20240430_S3408_M22sa71400gl_4	5.75E+05	1.98E+05	1.42E+06	4.90E+05	2.78E+05	9.60E+04	2.06E+00	2.18E-01	2.31E-01	5.10E+00	2.26E-01	2.07E-01	47.11	2.40E+00		42240	9.73E-01		40670
																Ave.	**40138**		Ave.	**41899**
																SD.	**1816**		SD.	**1001**
LVP-18	20240430_S3408_M30sa71450gl_1	7.80E+05	2.69E+05	1.20E+06	4.15E+05	3.63E+05	1.25E+05	2.15E+00	2.13E-01	2.01E-01	3.32E+00	2.24E-01	1.89E-01	46.29	1.54E+00		26978	9.94E-01		41576
	20240430_S3408_M30sa71450gl_2	7.54E+05	2.60E+05	1.18E+06	4.07E+05	3.57E+05	1.23E+05	2.11E+00	2.16E-01	2.03E-01	3.30E+00	2.03E-01	1.91E-01	46.29	1.53E+00		26868	9.77E-01		40844
	20240506_S3408_M30_sa71450gl_1	9.96E+05	3.44E+05	1.11E+06	3.83E+05	4.45E+05	1.53E+05	2.24E+00	2.00E-01	1.80E-01	2.50E+00	4.07E-01	1.77E-01	46.29	1.16E+00		23384	1.04E+00		38015
	20240506_S3408_M30_sa71450gl_2	9.77E+05	3.37E+05	1.15E+06	3.96E+05	4.40E+05	1.52E+05	2.22E+00	1.67E-01	1.81E-01	2.60E+00	2.94E-01	1.77E-01	46.29	1.21E+00		24400	1.03E+00		37653
	20240506_S3408_M30_sa71450gl_3	1.00E+06	3.46E+05	1.18E+06	4.07E+05	4.50E+05	1.55E+05	2.23E+00	1.64E-01	1.79E-01	2.62E+00	2.65E-01	1.75E-01	46.29	1.21E+00		24578	1.03E+00		37798
	20240506_S3408_M30_sa71450gl_4	9.89E+05	3.41E+05	1.16E+06	3.99E+05	4.45E+05	1.53E+05	2.22E+00	2.10E-01	1.81E-01	2.60E+00	3.24E-01	1.76E-01	46.29	1.20E+00		24358	1.03E+00		37754
																Ave.	**25095**		Ave.	**38940**
																SD.	**1477**		SD.	**153**
LVP-16	20240506_S3408_M36_noPt71600gl_1	5.04E+05	1.74E+05	2.17E+05	7.49E+04	5.30E+05	1.83E+05	9.52E-01	1.92E-01	1.97E-01	4.10E-01	4.45E-01	2.55E-01	54.51	2.05E-01		4517	4.76E-01		19290
	20240506_S3408_M36_noPt71600gl_2	4.94E+05	1.70E+05	2.10E+05	7.23E+04	5.16E+05	1.78E+05	9.56E-01	2.12E-01	1.99E-01	4.06E-01	4.47E-01	2.59E-01	54.51	2.03E-01		4476	4.78E-01		19386
																Ave.	**4496**		Ave.	**19338**
																SD.	**29**		SD.	**68**

H_2O and CO_2 contents are corrected for background by measuring Suparsil3002
Ave, average
SD, standard deviation
